# Supplementary material for: Potential Biocontrol Agents of Corn Tar Spot Disease Isolated from Overwintered Phyllachora maydis Stromata
Source: Microorganisms. 2023 Jun 10;11(6):1550. doi: 10.3390/microorganisms11061550 (PMC10303863; doi:10.3390/microorganisms11061550)
Supplement: Supplementary file 1 [file microorganisms-11-01550-s001.zip › Supplementary file S1.docx]

Supplementary Table S1. PCR primer pairs utilized in this study.

| Fungi | Gene | References |
| --- | --- | --- |
| V9D (5′-TTAAGTCCCTGCCCTTTGTA-3′) and LS266 (5′-GTAGTCATATGCTTGTCTC-3′) | Ribosomal DNA: partial small subunit (SSU), internal transcribed spacer 1 (ITS1), 5.8S subunit, ITS2, partial large subunit (LSU) | 43 |
| fRPB2-5F* (5’-GAYGAYMGWGATCAYTTYGG-3’)  fRPB2-7cR (5’-CCCATRGCTTGYTTRCCCAT-3’) | Partial RNA polymerase second largest subunit (*rpb2*) | 44 |
| Alt-for (5’-ATGCAGTTCACCACCATCGC-3’)  Alt-rev (5’-ACGAGGGTGAYGTAGGCGTC-3’) | Partial *Alternaria* major allergen (*Alt-a1*) | 45 |
| GPD1 (5’-CAACGGCTTCGGTCGCATTG-3’)  GPD2 (5’-GCCAAGCAGTTGGTTGTGC-3’) | Partial glyceraldehyde dehydrogenase (*gapdh*) | 46 |
| ACT 512F (5'-ATGTGCAAGGCCGGTTTCGC-3') ACT 783R (5'-TACGAGTCCTTCTGGCCCAT-3') | Partial actin | 47 |
| Bacteria |  |  |
| 27F (5′-AGAGTTTGATCCTGGCTCAG-3′) 1492R (5′-GGTTACCTTGTTACGACTT-3′) | *16S rDNA* | 48, 49 |
| UP1S (5'-GAAGTCATCATGACCGTTCTGCAYGCNGGNGGNAARTTYGA-3') UP2SR (5'-AGCAGGGTACGGATGTGCGAGCCRTCNACRTCNGCRTCNGTCAT-3') | Partial gyrase B *gyrB* | 50 |

*Sequencing results indicated the partial *rpb2* gene was not amplified using primers fRPB 5F/77cR and genomic DNA from *Alternaria* organism A. Therefore, the following primers were designed for amplifying the partial rpb2 gene only in *Alternaria* organism A:

Alter new RPB F 5’-AGAAGGAGGGCCAGGACACAC-3’

Alter new RPB R 5’-ACCCATAGCAGATTGGTAAGTGTTAC-3’
