# Supplementary material for: Potential Biocontrol Agents of Corn Tar Spot Disease Isolated from Overwintered Phyllachora maydis Stromata
Source: Microorganisms. 2023 Jun 10;11(6):1550. doi: 10.3390/microorganisms11061550 (PMC10303863; doi:10.3390/microorganisms11061550)
Supplement: Supplementary file 1 [file microorganisms-11-01550-s001.zip › Supplementary file S2.docx]

Partial gene sequences used to identify bacteria.

>1+ *Pseudomonas graminis, gyrB*

TACCCACTTCCTGGGACGAGATCATCTTGT

CGAAGCGAGCCTTTTCCACGTTCAGGATCTTGCCCTTGAGTGGCAGGATGGCCTGAGTCT

TACGGTTGCGTCCCTGCTTGGCGGAACCGCCAGCAGAGTCACCTTCCACAAGATACAGCT

CGGAAAGGGCAGGGTCCTTTTCCTGGCAGTCAGCCAGCTTGCCGGGCAGGCCTGCGATAT

CCAGCGCATCTTTGCGGCGAGTCATCTCACGGGCTTTACGCGCGGCTTCACGAGCACGTG

CCGCATCGATCATCTTGCC

>3 *Pantoea* *anthophila, gyrB*

AAGCTGGAGCTGACCATTCGTCGCGAAGGCAAAGTGCATCA

GCAGATTTACGTCCACGGCGTTCCCGAGTCGCCGCTGACCGTGACCGGCGATACCGATTT

AACCGGTACCCGCGTCCGTTTCTGGCCGAGCTACGAAACCTTCACCAACGTCCGCGATTT

TGAGTATGACATTCTGGCGAAACGCCTGCGTGAACTCTCCTTCCTGAACTCAGGCGTTTC

GATCCGTCTGGAAGATAAGCGTGACGGCAAAACGGATCACTTCCACTACGAAGGTGGTAT

CAAGGCGTTTGTTGAGTACCTGAACAAAAACAAAACCCCGATTCACCCGACCGTCTTCTA

CTTCTCAAACGAGAAAGATGGCATCGGTGTGGAAGTGGCGCTGCAGTGGAACGACGGTTT

CCAGGAAAATATCTACTGCTTTACCAACAACATCCCGCAGCGTGACGGCGGTACGCACCT

TGCCGGTTTCCGTGCGGCGATGACCCGTACCCTGAACGCCTACATGGATAAAGAGGGTTA

CAGCAAGAAAGCCAAAGTCAGCGCCACCGGTGACGATGCCCGTGAAGGCCTGATTGCCGT

CGTGTCGGTGAAAGTACCGGACCCTAAATTCTCCTCACAGACCAAAGA

>4 *Pantoea* *agglomerans, gyrB*

AGAAGCTGGAGCTGACCATTCGTCGCGAAGGCAAAGTGCATCAGC

AGATTTACGTCCATGGCGTACCTGAGGCACCGCTGGCAGTCACTGGTGATACCGATATCA

CCGGTACCCGCGTGCGTTTCTGGCCAAGCTATGAAACCTTTACCAACGTGCGCGATTTCG

AGTATGACATTCTGGCAAAACGCCTGCGCGAACTGTCGTTCCTGAACTCGGGCGTGTCGA

TTCGTCTGGAAGACAAGCGTGATGGCAAAACCGATCACTTCCATTACGAAGGCGGCATCA

AGGCGTTTGTTGAGTACCTCAACAAAAACAAAACCCCGATTCACCCTACCGTGTTCTATT

TCTCTACCGAGAAAGATGGCATTGGCGTGGAAGTAGCACTGCAGTGGAACGACGGCTTCC

AGGAAAATATTTACTGCTTTACCAACAACATTCCGCAGCGCGATGGCGGCACGCACCTTG

CCGGTTTCCGCGCAGCGATGACGCGTACCCTGAATGCCTACATGGATAAAGAGGGTTACA

GCAAGAAAGC

>5 *Pantoea* *ananatis, gyrB*

CAGAAACTGGAACTGACCATTCGTCGTGAAGGCAAAGTGCATCAGCA

GATTTATCTGCACGGCGTACCTGAAGCGCCGCTGGCGGTTACGGGAGATACCGATGCAAC

CGGTACCCGCATTCGTTTCTGGCCAAGCCATGACACCTTCACCAACGTTACCGATTTCGA

ATACGACATTCTGGCGAAACGCCTGCGCGAACTGTCCTTCCTGAACTCTGGCGTATCGAT

TCGTCTGGAAGACAAGCGTGATGCAAGAAACGACCACTTTCACTACGAAGGTGGTATCCG

CGCCTTTGTTGAGTACCTGAATAAAAACAAAACGCCTATTCACCCAACCGTATTCTATTT

CTCAACCGAGAAAGACGGCATTGGCGTGGAAGTCGCACTGCAGTGGAATGACGGCTTCCA

GGAGAACATCTACTGCTTCACGAACAATATTCCACAGCGCGACGGCGGTACACACCTTGC

GGGTTTCCGCGCCGCGATGACGCGTACGCTGAATGCCTACATGGATAAAGAAGGCTACAG

CAAAAAAGCCAAAGTCAGCGCGACCGGTGACGATGCCCGTGAAGGTCTGATTGCGG

>6 *Pseudomonas graminis, gyrB*

AAGAGATGTTGTGGTAGCGCAGTTTTTCGATGTTGTACTCGTCACGGCCAAT

ACCGCAGCCCAGTGCCGTGATCAACGTACCCACTTCCTGGGACGAGATCATCTTGTCGAA

GCGAGCCTTTTCCACGTTCAGGATCTTGCCCTTGAGTGGCAGGATGGCCTGAGTCTTACG

GTTGCGTCCCTGCTTGGCGGAACCGCCAGCAGAGTCACCTTCCACAAGATACAGCTCGGA

AAGGGCAGGGTCCTTTTCCTGGCAGTCAGCCAGCTTGCCGGGCAGGCCTGCGATATCCAG

CGCACCTTTGCGGCGAGTCATCTCACGGGCTTTACGCGCGGCTTCACGAGCACGTGCCGC

ATCGATCATCTTGCCGACAACGGCTTTCGCTTCGTTTGGGTTCTCGAGCAGGAAGTCAGA

AAAGTACTTGCCCATTTCCTGCTCAACCGCGGTCTTCACTTCTGAAGACACCAGCTTGTC

TTTCGTCTGCGAGCTGAACTTCGGATCAGGAACCTTGACGGAGATGATCGCTGTCAGGCC

TTCACGCGCATCGTCACCGGTGGTGGCGACCTTGTGCTTCTTCGCCAGACCTTCCTGCTC

AATGTAGTTGTTCAGGTTACGTGTCA

>7 *Pantoea eucalypti, gyr B*

AAGTCTCCGGCGGCCTGCATGGCGTGGGCGTCTCCGTGGTTAACGCCCTG

TCGCAGAAGCTGGAGCTGACCATTCGTCGCGAAGGCAAAGTGCATCAGCAGGTTTACGTC

CATGGCGTACCGCAGTCACCGCTGGCGGTAACGGGTGAAACCGACATTACCGGGACCCGC

GTGCGTTTCTGGCCGAGCCACGAAACCTTTACCAACGTGCGCGATTTTGAGTATGACATT

CTGGCGAAACGCCTGCGTGAACTCTCATTCCTCAACTCGGGCGTGTCTATTCGTCTGGAA

GACAAGCGCGATGGCAAAAACGATCACTTCCATTACGAAGGCGGCATCCGGGCGTTTGTT

GAGTACCTGAACAAAAACAAAACCCCTATTCACCCTACCGTGTTCTATTTCTCTACTGAG

AAAGATGGCATCGGTGTGGAAGTGGCACTGCAGTGGAACGACGGTTTCCAGGAAAACATC

TACTGCTTTACCAACAACATCCCGCAGCGTGACGGTGGTACCCACCTCGCCGGTTTCCGT

GCGGCGATGACCCGTACCCTGAATGCCTACATGGACAAAGAGGGTTACAGCAAGAAAGCC

AAAGTCAGCGCCACCGGTGATGATGCCCGTGAAGGTCTGATCGCCGTAGTGTCGGTGAAA

GTGCCGGATCCAAAATTCTCCTCGCAGACCAAAGATAAACTGGTCTCCTCCGAGGTGAAA

TCGGCGGTTGAGCAGCAGATGAATGAGCTGCTGGCAGAATACCTGCTGGAAAATCCATCA

>10B *Priestia* *megaterium, gyrB*

TAAAGGCAAAATAGCTTGGAAATG

ACGGCTTCTTCCCTGCTTAGCTGAACCCCCGGCAGAGTCACCCTCTACTACATAGAGTTC

GCTAATAGAAGGATCTTTTGATGAACAATCTGCTAATTTACCCGGTAAGTTTGAGATTTC

AAGCGCGCTTTTACGTCTTGTAAGCTCACGAGCTTTTTTAGCTGCCATTCTTGCTCTTGC

AGCCATTAAACCTTTTTCAATTACCTTTTTCGCCACAATAGGGTTCTCTAGCAAGTACGT

TTCTAAGTGTTCTGCAAACACAGAGTCAGTAATTGTTCTTGCTTCACTATTTCCCAGCTT

TGTTTTTGTTTGTCCTTCGAACTGCGGATCTGGGTGCTTAATAGAGATGATAGCTGTAAT

TCCTTCACGAACATCTTCACCCGTTAGATTGGCGTCACTGTCTTTAAATACGCTGTTTTT

ACGTGCATAGTCGTTAATTACACGCGTTAACGCTGTTTTAAATCCTGCTTCGTGTGTTCC

ACCTTC

>10 *Curtobacterium flaccumfaciens, gyrB*

GTACGGGCCTTGTCCGGGTCGAAGTCCTCACCGAT

GCCGGCACCGAACGCCGTGATCATCGACTGGATCTCCTGGTTGGCGAGGGCACGGTCGAG

CCGGGCCTTCTCGACGTTCAGGATCTTGCCGCGCAGGGGCAGGATCGCCTGGGTCATCGG

GTTGCGGCCCTGCACGGCGGAACCGCCGGCGGAGTCACCCTCGACCATGAAGATCTCCGA

CACCGTCGGGTCCTTCGACTGGCAGTCCTTGAGCTTGCCGGGCATGCCGCCCGACTCGAG

CAGCCCCTTGCGGCGGGTGGTCTCGCGCGCCTTGCGGGCGGCCAGACGTGCCTGCGACGC

CTGGATCGCCTTGCGGACGACGTCGCGTGCCTGCGTCGGGTTGCTCTCGAACCAGTGGGT

CAGCTCGGTGCCGACGACGCGCTGCACGAACGACTTCGCCTCGGTGTTGCCGAGCTTGGT

CTTCGTCTGGCCCTCGAACTGCGGCTCGCCGAGCTTGACGGA

>11 *Priestia* *megaterium,* *gyrB*

AATGATTGTACGAATTTCATTATTAGATAAAATTTTATCTA

AACGCGCTTTCTCTACGTTGATAATTTTACCACGTAAAGGCAAAATAGCTTGGAAATGAC

GGCTTCTTCCCTGCTTAGCTGAACCCCCGGCAGAGTCACCCTCTACTACATAGAGTTCGC

TAATAGAAGGATCTTTTGATGAACAATCTGCTAATTTACCCGGTAAGTTTGAGATTTCAA

GCGCGCTTTTACGTCTTGTAAGCTCACGAGCTTTTTTAGCTGCCATTCTTGCTCTTGCAG

CCATTAAACCTTTTTCAATTACCTTTTTCGCCACAATAGGGTTCTCTAACAAGTACGTTT

CTAAGTGTTCTGCAAACACAGAGTCAGTAATTGTTCTTGCTTCACTATTTCCCAGCTTTG

TTTTTGTTTGTCCTTCGAACTGCGGATCTGGGTGCTTAATAGAGATGATAGCTGTAATTC

CTTCACGAACATCTTCACCCGTTAGATTGGCGTCACTGTCTTTAAATACGCTGTTTTTAC

GTGCATAGTCGTTAATTACACGCGTTAACGCTGTTTTAAATCCTGCTTCGTGTGTTCCAC

CTTCATATGTGTGAATATTG

>12 *Pseudomonas graminis, gyrB*

TGCCGTGATCAACGTACCCACTTCCTGGGACGAGATC

ATCTTGTCGAAGCGAGCCTTTTCCACGTTCAGGATCTTGCCCTTGAGTGGCAGGATGGCC

TGAGTCTTACGGTTGCGTCCCTGCTTGGCGGAACCGCCAGCAGAGTCACCTTCCACAAGA

TACAGCTCGGAAAGGGCAGGGTCCTTTTCCTGGCAGTCAGCCAGCTTGCCGGGCAGGCCT

GCGATATCCAGCGCACCTTTGCGGCGAGTCATCTCACGGGCTTTACGCGCGGCTTCACGA

GCACGTGCCGCATCGATCATCTTGCCGACAACGGCTTTCGCTTCGTTTGGGTTCTCGAGC

AGGAAGTCAGAAAAGTACTTGCCCATTTCCTGCTCAACCGCGGTCTTCACTTCTGAAGAC

ACCAGCTTGTCTTTCGTCTGCGAGCTGAACTTCGGATCAGGAACCTTGACGGAGATGATC

GCTGTCAGGCCTTCACGCGCATCGTCACCGGTGGTGGCGACCTTGTGCTTCTTCGCCAGA

CCTTCCTGCTCAATGTA

>13 *Curtobacterium flaccumfaciens, gyrB*

GGGCCTTGTCCGGGTCGAAGTCCTCACCGATG

CCGGCACCGAACGCCGTGATCATCGACTGGATCTCCTGGTTGGCGAGGGCGCGGTCGAGC

CGGGCCTTCTCGACGTTCAGGATCTTGCCGCGCAGGGGCAGGATCGCCTGGGTCATCGGG

TTGCGGCCCTGCACGGCGGAACCGCCAGCGGAGTCACCCTCGACCATGAAGATCTCCGAC

ACGGTCGGGTCCTTCGACTGGCAGTCCTTGAGCTTGCCGGGCATGCCGCCCGACTCGAGC

AGCCCCTTGCGGCGGGTGGTCTCGCGCGCCTTGCGGGCAGCGAGCCGCGCCTGCGACGCC

TGGATCGCCTTGCGGACGACGTCGCGTGCCTGCGTCGGGTTGCTCTCGAACCAGTGGGTC

AGCTCGGTGCCGACGACGCGCTGCACGAACGACTTCGCCTCGGTGTTGCCGAGCTTGGTC

TTCGTCTGCCCCTCGAACTGCGGCTCGCCGAGCTTCACGGAGATGACGGCCGTCAGCCCT

TCGCGGATGTCGTCACCCGTGAGGTTGTCGTCCTTCTCCTTGATGATCTTCGTGTCGCGC

GCGTAACGGTTGACGAGGGTCGTGAGCGCTGCACGGAA

>14 *Pseudomonas* *prosekii 16S rDNA*

GCCTACCATGCAAGTCGAGCGGTAGAGAGAAGCTTGCTTCTCTTGAGAGCGGC

GGACGGGTGAGTAATGCCTAGGAATCTGCCTGGTAGTGGGGGATAACGCTCGGAAACGGA

CGCTAATACCGCATACGTCCTACGGGAGAAAGCAGGGGACCTTCGGGCCTTGCGCTATCA

GATGAGCCTAGGTCGGATTAGCTAGTTGGTGAGGTAATGGCTCACCAAGGCGACGATCCG

TAACTGGTCTGAGAGGATGATCAGTCACACTGGAACTGAGACACGGTCCAGACTCCTACG

GGAGGCAGCAGTGGGGAATATTGGACAATGGGCGAAAGCCTGATCCAGCCATGCCGCGTG

TGTGAAGAAGGTCTTCGGATTGTAAAGCACTTTAAGTTGGGAGGAAGGGCAGTTACCTAA

TACGTATCTGTTTTGACGTTACCGACAGAATAAGCACCGGCTAACTCTGTGCCAGCAGCC

GCGGTAATACAGAGGGTGCAAGCGTTAATCGGAATTACTGGGCGTAAAGCGCGCGTAGGT

GGTTTGTTAAGTTGGATGTGAAATCCCCGGGCTCAACCTGGGAACTGCATTCAAAACTGA

CAAGCTAGAGTATGGTAGAGGGTGGTGGAATTTCCTGTGTA

>F1 *Pantoea* *eucalypti, gyrB*

CGGCGGCCTGCATGGCGTGGGCGTCTCCGTGGTTAACGCCCTGT

CGCAGAAGCTGGAGCTGACCATTCGTCGCGAAGGCAAAGTGCATCAGCAGGTTTACGTCC

ATGGCGTACCGCAGTCACCGCTGGCGGTAACGGGTGAAACCGACATTACCGGGACCCGCG

TGCGTTTCTGGCCGAGCCACGAAACCTTTACCAACGTGCGCGATTTTGAGTATGACATTC

TGGCGAAACGCCTGCGTGAACTCTCATTCCTCAACTCGGGCGTGTCTATTCGTCTGGAAG

ACAAGCGCGATGGCAAAAACGATCACTTCCATTACGAAGGCGGCATCCGGGCGTTTGTTG

AGTACCTGAACAAAAACAAAACCCCTATTCACCCTACCGTGTTCTATTTCTCTACTGAGA

AAGATGGCATCGGTGTGGAAGTGGCACTGCAGTGGAACGACGGTTTCCAGGAAAACATCT

ACTGCTTTACCAACAACATCCCGCAGCGTGACGGTGGTACCCACCTCGCCGGTTTCCGTG

CGGCGATGACCCGTACCCTGAATGCCTACATGGACAAAGAGGGTTACAGCAAGAAAGCCA

AAGTCAGCGCCACCGGTGATGATGCCCGTGAAGGTCTGATCGCCGTAGTGTCGGTGAAAG

TGCCGGATCCAAAATTCTCCTCGCAGACCAAAGATAAACTGGTCTCCTCCGAGGTGAAAT

CGGCGGTTGAGCA

>F2 *Pantoea agglomerans, gyrB*

GCTGGAGCTGACCATTCGTCGCGAAGGCAAAGTGCATC

AGCAGGTTTACGTCCATGGCGTACCGCAGGCACCGCTGGCGGTAACGGGTGAGACAGACA

TTACCGGGACCCGTGTGCGTTTCTGGCCGAGCCACGAAACCTTTACCAACGTGCGCGATT

TTGAGTATGACATTCTGGCGAAACGCCTGCGTGAACTCTCATTCCTCAACTCGGGCGTGT

CTATTCGTCTGGAAGATAAGCGCGATGGCAAAAACGATCACTTCCATTACGAAGGCGGCA

TCCGGGCGTTTGTTGAGTACCTGAACAAAAACAAAACCCCTATTCACCCTACCGTGTTCT

ATTTCTCTACTGAGAAAGATGGCATCGGTGTGGAAGTGGCACTGCAGTGGAACGACGGTT

TCCAGGAAAACATCTACTGCTTTACCAACAACATCCCGCA

>F3 *Pseudomonas quercus, 16S rDNA*

TCGCGCCTCCATGCAAGTCGAGCGGCAGCACGGGAGCTTGCTCCTGGTGGCGAGCGGCGG

ACGGGTGAGTAATGCCTAGGAATCTGCCTGATAGTGGGGGATAACAACCGGAAACGGTTG

CTAATACCGCATACGTCCTACGGGAGAAAGTGGGGGACCTTCGGGCCTCACGCTATCAGA

TGAGCCTAGGTCGGATTAGCTAGTTGGTAGGGTAAAGGCCTACCAAGGCGACGATCCGTA

ACTGGTCTGAGAGGATGATCAGTCACACTGGAACTGAGACACGGTCCAGACTCCTACGGG

AGGCAGCAGTGGGGAATATTGGACAATGGGCGAAAGCCTGATCCAGCCATGCCGCGTGTG

TGAAGAAGGTCTTCGGATTGTAAAGCACTTTAAGTTGGGAGGAAGGGCAGTCAGCGAATA

CCTGGTTGTCTTGACGTTACCGACAGAATAAGCACCGGCTAACTCTGTGCCAGCAGCCGC

GGTAATACAGAGGGTGCAAGCGTTAATCGGAATTACTGGGCGTAAAGCGCGCGTAGGCGG

TTTGTCAAGTCGAATGTGAAATCCCCGGGCTCAACCTGGGAACTGCATCCGAAACTGGCA

AGCTAGAGTATGGTAGAGGGTGGTGGAATTTCCTGTGTAGCGGTGAAATGCGTAGATATA

GGAAGGAACATCAGTGGCGAAGGCGACCACCTGGACCAATACTGACGCTGAGGTGCGAAA

GCGTGGGGAGCAAACAGGATTAGATACCCTGGTAGTCCACGCCGTAAACGATGTCAACTA

GCCGTTGGGATCCTTGAGATCTTAGTGGCGCAGCTAACGCATTAAGTTGACCGCCTGGGG

AGTACGGCCGCAAGGTTAAAACTCAAATGAATTGACGGGGGCCCGCACAAGCGGTGGA

>F5 *Pantoea* *agglomerans, gyrB*

AGAAGCTGGAGCTGACCATTCGTCGCGAAGGCAAAGTGCATCAGCA

GATTTACGTCCATGGCGTACCTGAGGCACCGCTGGCAGTCACTGGTGATACCGATATCAC

CGGTACCCGCGTGCGTTTCTGGCCAAGCTATGAAACCTTTACCAACGTGCGCGATTTCGA

GTATGACATTCTGGCAAAACGCCTGCGCGAACTGTCGTTCCTGAACTCGGGCGTGTCGAT

TCGTCTGGAAGACAAGCGTGATGGCAAAACCGATCACTTCCATTACGAAGGCGGCATCAA

GGCGTTTGTTGAGTACCTCAACAAAAACAAAACCCCGATTCACCCTACCGTGTTCTATTT

CTCTACCGAGAAAGATGGCATTGGCGTGGAAGTAGCACTGCAGTGGAACGACGGCTTCCA

GGAAAATATTTACTGCTTTACAAACAACATTCCGCAGCGCGATGGCGGTACGCACCTTGC

CGGTTTCCGCGCAGCGATGACGCGTACCCTGAATGCCTACATGGATAAAGAGGGTTACAG

CAAGAAAGCCAAAGTCAGCGCCACCGGTGATGATGCGCGTGAAGGTCTGATCGCCG

>E2 *Pseudomonas fluorescens* or *shahriarae, gyrB*

GCGACGGTTGCGACCCTGCTTGGCGGAACCGCCAGCAGAGTCAC

CTTCCACCAAGTACAGTTCGGAAAGGGCAGGGTCCTTCTCCTGGCAGTCCGCCAGTTTGC

CCGGCAGGCCAGCGATATCCAGTGCACCTTTACGGCGGGTCATTTCACGGGCTTTACGCG

CCGCTTCACGAGCCCGTGCCGCGTCGATCATCTTGCCGACGACCAGCTTGGCTTCGTTGG

GGTTTTCCAGCAGGAAGTCGGAGAAGTATTTGCCCATTTCCTGTTCGACGGCGGTCTTCA

CTTCAGA

>E3 *Priestia* *flexa, gyrB*

CACGCCCGCCTATTACAGGTTTGTGATAACGGTGCCTTAGAAATATCAAAATCTTCTCCA

ATACCTGGTACCTAATGCCGTGATAATTGTACGAATCTCGTTATTAGATAAAATTTTATC

TAGTCTTGCTTTTTCAACGTTAATAATTTTACCACGCAAAGGTAGGATAGCTTGGAAATG

ACGACTACGCCCTTGTTTAGCAGAACCACCTGCAGAATCACCCTCTACAATGTACAATTC

ACTGATGGAAGGATCCTTTGAAGAGCAATCTGCTAATTTACCAGGTAGATTTGAGATCTC

CAATGCGCTTTTTCTTCTTGTTAATTCTCTTGCTTTTTTAGCTGCCATTCGAGCACGTGC

TGCCATAACACCTTTTTCAACAATTTTCTTAGCTACTACTGGATTTTCTAGTAGGTAAGT

TTCAAAGTGCTCTGCAAATACAGAATCAGTAATAGTTCTTGCTTCACTATTCCCTAATTT

TGTTTTTGTTTGACCTTCAAATTGAGGGTCTGGATGTTTAATTGAAATGATTGCAGTAAT

TCCCTCACGTACATCTTCACCTGTAAGG

Partial gene sequences used to identify fungi

>E9F *Cladosporium* *crousii* (actin sequence)

TTATGTGCAAGGGCCGGTTTCGCCGGTGACGATGCGCCCAGAGCCGTTTTCCGTAAGTCT

AAAGACACCTGCTTCGCCCGCGCCAGAATCCCG

AGCTGACACCCTTCTCTAGCTTTCCATNGNTCGGCAGACCCCGNTCACCATGGGTATGCA

TTCTCCCCGCGAGCCTCCCATTCGCGCTCATTCAAATCTAACCCCGGCACAGATATCATG

ATCGGTATGGGCCAGAAAGGACTCGTA

>10RDF *Cladosporium* *rectoides* (actin sequence)

TTATGTGCAAGGNCGGTTTCGCCGGTGACGATGCGCCCAGAGCCGTTTTCCGTAAGTCTA

AAGACACCTGTTTCGCCCATCCCGCAATT

CCGAGCTGACACCCANTTTTAGCNTTCCATTGTCGGCAGACCCCGNTCACCATGGGTATG

CATTTCCCCCTGCGAACCTCCCTATCGCGCTCAACCGTGTCTAACCCCGGCGCAGTATCA

TGATCGGTATGGGCCAGAAGGACTCGTA

>13RPF *Cladosporium* *subuliforme* (actin sequence)

TTATGTGCAAGGCCGGTTTCGCCGGTGACGATGCGCCCAGAGCCGTTTTCCGTAAGTCTG

AAGACACCTGTTTCGCCCATCCCGCAATTCCCA

GCTGACACTCCTTTNCAGCATTCCATTGTCGGCAGACCCCGNTCACCATGGGTATGCATT

TTCCCTGCGATCCCCTCTATCGCGCGCGGCCAATTCTAACCCCTCCGCAGTATCATGATC

GGTATGGGCCAGAAGGACTCGTA

>11C-F *Alternaria alternata/arborescens*, *Alt-a1* gene

CGCCTCTCTCTTCGCCGCCGCTGGCCTTGCCGCTGCTGCTCCTCTCGAGTCTCGCCAGGA

CACCGCATCCTGCCCTGTCACCACTGAGGGTGACTACGTCTGGAAGATCTCCGAATTCTA

CGGACGCAAGCCGGAAGGAACCTACTACAACAGCCTCGGCTTCAACATCAAGGCCACCAA

CGGAGGAACCCTCGACTTCACCTGCTCTGCTCAGGCCGATAAGCTTGAGGACCACAAGTG

GTACTCCTGTGGCGAGAACAGCTTCATGGACTTCTCTTTCGACAGCGACCGCAGCGGTCT

GCTCCTGAAGCAGAAGGTTAGCGACGAGTAAGTTACCCTTGTACCTTCGATTACTTCGCA

GATTCAGATATACTAACATATTCCCAGCATCACCTATGTCGCTACCGCCACTCTTCCCAA

CTACTGCCGCGCTGGCGGTAACGGCCCTAAGGACTTTGTCTGCCAGGGTGTTGC

>14C-F *Alternaria alternata/arborescens*, *Alt-a1* gene

ATCGCCTCTCTCTTCGCCGCCGCTGGCCTTGCCGCTGCTGCTCCTCTCGAGTCTCGCCAG

GACACCGCATCCTGCCCTGTCACCACTGAGGGTGACTACGTCTGGAAGATCTCCGAATTC

TACGGACGCAAGCCGGAAGGAACCTACTACAACAGCCTCGGCTTCAACATCAAGGCCACC

AACGGAGGAACCCTCGACTTCACCTGCTCTGCTCAGGCCGATAAGCTTGAGGACCACAAG

TGGTACTCCTGTGGCGAGAACAGCTTCATGGACTTCTCTTTCGACAGCGACCGCAGCGGT

CTGCTCCTGAAGCAGAAGGTTAGCGACGAGTAAGCTACCCTTGTACCTTCGATTACTTCG

CAGATTCAGATATACTAACATATTCCCAGCATCACCTATGTCGCTACCGCCACTCTTCCC

AACTACTGCCGCGCTGGCGGTAACGGCCCTAAGGACTTTGTCTGCCAGGGTGTTGC

>15F *Alternaria alternata/arborescens*, *Alt-a1* gene

GCCTCTCTNTTCGCCGCCGCTGGCCTTGCCGCTGCTGCTCCTCTCGAGTCTCGCCAGGAC

ACCGCATCCTGCCCTGTCACCACTGAGGGTGACTACGTCTGGAAGATCTCCGAATTCTAC

GGACGCAAGCCGGAAGGAACCTACTACAACAGCCTCGGCTTCAACATCAAGGCCACCAAC

GGAGGAACCCTCGACTTCACCTGCTCTGCTCAGGCCGATAAGCTTGAGGACCACAAGTGG

TACTCCTGTGGCGAGAACAGCTTCATGGACTTCTCTTTCGACAGCGACCGCAGCGGTCTG

CTCCTGAAGCAGAAGGTTAGCGACGAGTAAGTTACCCTTGTACCTTCGATTACTTCGCAG

ATTCAGATATACTAACATATTCCCAGCATCACCTATGTCGCTACCGCCACTCTTCCCAAC

TACTGCCGCGCTGGCGGTAACGGCCCTAAGGACTTTGTCTGCCAGGGTGTTGCCGA

>1C-F *Alternaria ovoidea*, *Alt-a1* gene

CCTCTCTCTTCGCCGCCGCTGGCCTTGCCGCCGCTGCACCTCTCGAGTCTCGCCAGGACA

CCGCATCCTGCCCTGTCACCACCGAGGGTGACTACGTCTGGAAGATTTCCGAGTTCTACG

GACGCAAGCCGGAGGGAACCTACTACAACAGCCTCGGCTTCAACATCAAGGCTACCAACG

GAGGAACACTCGACTTCACCTGCTCTCACTCAGCCGACAAGCTTGAGGACCACACTTGGT

ACTCTTGCGGCGAGAACAGCTTCATGGACTTCTCTTTCGACAGCGACCGCAACGGTCTGC

TCCTGAAGCAGAAGGTTAGCGACGAGTAAGTTACCCTTGTACCTTCGATTACTTCGCAGA

TTCAGATATACTAACATGTTTCCAGCATCACCTATGTCGCTACCGCCACTCTTCCCAACT

ACTGCCGCGCTGGCGGTAACGGCCCTAAGGACTTTGTCTGCCAGGGTGTTGCCGA

>2-F *Alternaria alternata/arborescens*, *Alt-a1* gene

TCGCCTCTCTCTTCGCCGCCGCTGGCCCTTGCCGCTGCTGCTCCTCTCGAGTCTCGCCAG

GACACCGCATCCTGCCCTGTCACCACTGAGGGTGACTACGTCTGGAAGATCTCCGAATTC

TACGGACGCAAGCCGGAAGGAACCTACTACAACAGCCTCGGCTTCAACATCAAGGCCACC

AACGGAGGAACCCTCGACTTCACCTGCTCTGCTCAGGCCGATAAGCTTGAGGACCACAAG

TGGTACTCCTGTGGCGAGAACAGCTTCATGGACTTCTCTTTCGACAGCGACCGCAGCGGT

CTGCTCCTGAAGCAGAAGGTTAGCGACGAGTAAGTTACCCTTGTACCTTCGATTACTTCG

CAGATTCAGATATACTAACATATTCCCAGCATCACCTATGTCGCTACCGCCACTCTTCCC

AACTACTGCCGCGCTGGCGGTAACGGCCCTAAGGACTTTGTCTGCCAGGGTGTTGCCGA

>3-CF *Alternaria ovoidea*, *Alt-a1* gene

GCCTCTCTNTTCGCCGCCGCTGGCCTTGCCGCCGCTGCACCTCTCGAGTCTCGCCAGGAC

ACCGCATCCTGCCCTGTCACCACCGAGGGTGACTACGTCTGGAAGATTTCCGAGTTCTAC

GGACGCAAGCCGGAGGGAACCTACTACAACAGCCTCGGCTTCAACATCAAGGCTACCAAC

GGAGGAACACTCGACTTCACCTGCTCTCACTCAGCCGACAAGCTTGAGGACCACACTTGG

TACTCTTGCGGCGAGAACAGCTTCATGGACTTCTCTTTCGACAGCGACCGCAACGGTCTG

CTCCTGAAGCAGAAGGTTAGCGACGAGTAAGTTACCCTTGTACCTTCGATTACTTCGCAG

ATTCAGATATACTAACATGTTTCCAGCATCACCTATGTCGCTACCGCCACTCTTCCCAAC

TACTGCCGCGCTGGCGGTAACGGCCCTAAGGACTTTGTCTGCCAGGGTGTTGCCGA

>4C-F *Alternaria alternata/arborescens*, *Alt-a1* gene

CTCTCTCTTCGCCGCCGCTGGCCTTGCCGCTGCTGCTCCTCTCGAGTCTCGCCAGGACAC

CGCATCCTGCCCTGTCACCACTGAGGGTGACTACGTCTGGAAGATCTCCGAATTCTACGG

ACGCAAGCCGGAAGGAACCTACTACAACAGCCTCGGCTTCAACATCAAGGCCACCAACGG

AGGAACCCTCGACTTCACCTGCTCTGCTCAGGCCGATAAGCTTGAGGACCACAAGTGGTA

CTCCTGTGGCGAGAACAGCTTCATGGACTTCTCTTTCGACAGCGACCGCAGCGGTCTGCT

CCTGAAGCAGAAGGTTAGCGACGAGTAAGTTACCCTTGTACCTTCGATTACTTCGCAGAT

TCAGATATACTAACATATTCCCAGCATCACCTATGTCGCTACCGCCACTCTTCCCAACTA

CTGCCGCGCTGGCGGTAACGGCCCTAAGGACTTTGTCTGCCAGGGTGTTGCCG

>5 *Alternaria alternata/arborescens*, *Alt-a1* gene

GCCTCTCTCTTCGCCGCCGCTGGCCTTGCCGCTGCTGCTCCTCTCGAGTCTCGCCAGGAC

ACCGCATCCTGCCCTGTCACCACTGAGGGTGACTACGTCTGGAAGATCTCCGAATTCTAC

GGACGCAAGCCGGAAGGAACCTACTACAACAGCCTCGGCTTCAACATCAAGGCCACCAAC

GGAGGAACCCTCGACTTCACCTGCTCTGCTCAGGCCGATAAGCTTGAGGACCACAAGTGG

TACTCCTGTGGCGAGAACAGCTTCATGGACTTCTCTTTCGACAGCGACCGCAGCGGTCTG

CTCCTGAAGCAGAAGGTTAGCGACGAGTAAGTTACCCTTGTACCTTCGATTACTTCGCAG

ATTCAGATATACTAACATATTCCCAGCATCACCTATGTCGCTACCGCCACTCTTCCCAAC

TACTGCCGCGCTGGCGGTAACGGCCCTAAGGACTTTGTCTGCCAGGGTGTTGCCG

>5C *Alternaria ovoidea*, *Alt-a1* gene

CGCCTCTCTCTTCGCCGCCGCTGGCCTTGCCGCCGCTGCACCTCTCGAGTCTCGCCAGGA

CACCGCATCCTGCCCTGTCACCACCGAGGGTGACTACGTCTGGAAGATTTCCGAGTTCTA

CGGACGCAAGCCGGAGGGAACCTACTACAACAGCCTCGGCTTCAACATCAAGGCTACCAA

CGGAGGAACACTCGACTTCACCTGCTCTCACTCAGCCGACAAGCTTGAGGACCACACTTG

GTACTCTTGCGGCGAGAACAGCTTCATGGACTTCTCTTTCGACAGCGACCGCAACGGTCT

GCTCCTGAAGCAGAAGGTTAGCGACGAGTAAGTTACCCTTGTACCTTCGATTACTTCGCA

GATTCAGATATACTAACATGTTTCCAGCATCACCTATGTCGCTACCGCCACTCTTCCCAA

CTACTGCCGCGCTGGCGGTAACGGCCCTAAGGACTTTGTCTGCCAGGGTGTTGCCG

>6 *Alternaria alternata/arborescens*, *Alt-a1* gene

CGCCTCTCTCTTCGCCGCCGCTGGCCTTGCCGCTGCTGCTCCTCTCGAGTCTCGCCAGGA

CACCGCATCCTGCCCTGTCACCACTGAGGGTGACTACGTCTGGAAGATCTCCGAATTCTA

CGGACGCAAGCCGGAAGGAACCTACTACAACAGCCTCGGCTTCAACATCAAGGCCACCAA

CGGAGGAACCCTCGACTTCACCTGCTCTGCTCAGGCCGATAAGCTTGAGGACCACAAGTG

GTACTCCTGTGGCGAGAACAGCTTCATGGACTTCTCTTTCGACAGCGACCGCAGCGGTCT

GCTCCTGAAGCAGAAGGTTAGCGACGAGTAAGTTACCCTTGTACCTTCGATTACTTCGCA

GATTCAGATATACTAACATATTCCCAGCATCACCTATGTCGCTACCGCCACTCTTCCCAA

CTACTGCCGCGCTGGCGGTAACGGCCCTAAGGACTTTGTCTGCC

>6C *Alternaria ovoidea*, *Alt-a1* gene

GCCTCTCTCTTCGCCGCCGCTGGCCTTGCCGCCGCTGCACCTCTCGAGTCTCGCCAGGAC

ACCGCATCCTGCCCTGTCACCACCGAGGGTGACTACGTCTGGAAGATTTCCGAGTTCTAC

GGACGCAAGCCGGAGGGAACCTACTACAACAGCCTCGGCTTCAACATCAAGGCTACTAAC

GGAGGAACACTCGACTTCACCTGCTCTCACTCAGCCGACAAGCTTGAGGACCACACTTGG

TACTCTTGCGGCGAGAACAGCTTCATGGACTTCTCTTTCGACAGCGACCGCAACGGTCTG

CTCCTGAAGCAGAAGGTTAGCGACGAGTAAGTTACCCTTGTACCTTCGATTACTTCGCAG

ATTCAGATATACTAACATGTTTCCAGCATCACCTATGTCGCTACCGCCACTCTTCCCAAC

TACTGCCGCGCTGGCGGTAACGGCCCTAAGGACTTTGTCTGCCAGGGTGTTGCC

>7 *Alternaria alternata/arborescens*, *Alt-a1* gene

CGCCTCTCTNTTCGCCGCCGCTGGCCTTGCCGCTGCTGCTCCTCTCGAGTCTCGCCAGGA

CACCGCATCCTGCCCTGTCACCACTGAGGGTGACTACGTCTGGAAGATCTCCGAATTCTA

CGGACGCAAGCCGGAAGGAACCTACTACAACAGCCTCGGCTTCAACATCAAGGCCACCAA

CGGAGGAACCCTCGACTTCACCTGCTCTGCTCAGGCCGATAAGCTTGAGGACCACAAGTG

GTACTCCTGTGGCGAGAACAGCTTCATGGACTTCTCTTTCGACAGCGACCGCAGCGGTCT

GCTCCTGAAGCAGAAGGTTAGCGACGAGTAAGTTACCCTTGTACCTTCGATTACTTCGCA

GATTCAGATATACTAACATATTCCCAGCATCACCTATGTCGCTACCGCCACTCTTCCCAA

CTACTGCCGCGCTGGCGGTAACGGCCCTAAGGACTTTGTCTGCCAGGGTGTTGCCG

>7C *Alternaria* species, *Alt-a1* gene

CCTCTCTNTTCGCCGCCGCTGGCCTTGCCGCCGCTGCTCCTCTCGAGTCTCGCCAGGACA

CCGCATCCTGCCCTGTCACCACCGAGGGTGACTACGTCTGGAAGATCTCCGAGTTCTACG

GACGCAAGCCGGAGGGAACCTACTACAACAGCCTCGGCTTCAACATCAAGGCTACCAACG

GAGGAACACTCGACTTCACCTGCTCTCACTCAGCCGACAAGCTTGAGGACCACACTTGGT

ACTCTTGCGGCGAGAACAGCTTCATGGACTTCTCTTTCGACAGCGACCGCAACGGTCTGC

TCCTGAAGCAGAAGGTTAGCGACGAGTAAGTTACCCTTGTACCTTCGATTACTTCGCAGA

TTCAGATATACTAACATATTCCCAGCATCACCTATGTCGCTACCGCCACTCTTCCCAACT

ACTGCCGCGCTGGCGGTAACGGCCCTAAGGACTTTGTCTGCCAGGGTGTTGCCGAC

>8 *Alternaria alternata/arborescens*, *Alt-a1* gene

CGTTTCTCTNTTCGCCGCCGCTGGCCTTGCCGCTGCTGCTCCTCTCGAGTCTCGCCAGGA

CACCGCATCCTGCCCTGTCACCACTGAGGGTGACTACGTCTGGAAGATCTCCGAATTCTA

CGGACGCAAGCCGGAAGGAACCTACTACAACAGCCTCGGCTTCAACATCAAGGCCACCAA

CGGAGGAACCCTCGACTTCACCTGCTCTGCTCAGGCCGATAAGCTTGAGGACCACAAGTG

GTACTCCTGTGGCGAGAACAGCTTCATGGACTTCTCTTTCGACAGCGACCGCAGCGGTCT

GCTCCTGAAGCAGAAGGTTAGCGACGAGTAAGTTACCCTTGTACCTTCGATTACTTCGCA

GATTCAGATATACTAACATATTCCCAGCATCACCTATGTCGCTACCGCCACTCTTCCCAA

CTACTGCCGCGCTGGCGGTAACGGCCCTAAGGACTTTGTCTGCCAGGGTGTTGCCG

>8-C *Alternaria alternata/arborescens*, *Alt-a1* gene

TCGCCTCTCTCTTCGCCGCCGCTGGCCTTGCCGCTGCTGCTCCTCTCGAGTCTCGCCAGG

ACACCGCATCCTGCCCTGTCACCACTGAGGGTGACTACGTCTGGAAGATCTCCGAATTCT

ACGGACGCAAGCCGGAAGGAACCTACTACAACAGCCTCGGCTTCAACATCAAGGCCACCA

ACGGAGGAACCCTCGACTTCACCTGCTCTGCTCAGGCCGATAAGCTTGAGGACCACAAGT

GGTACTCCTGTGGCGAGAACAGCTTCATGGACTTCTCTTTCGACAGCGACCGCAGCGGTC

TGCTCCTGAAGCAGAAGGTTAGCGACGAGTAAGTTACCCTTGTACCTTCGATTACTTCGC

AGATTCAGATATACTAACATATTCCCAGCATCACCTATGTCGCTACCGCCACTCTTCCCA

ACTACTGCCGCGCTGGCGGTAACGGCCCTAAGGACTTTGTCTGCCAGGGTGTTGCCG

>9-F *Alternaria alternata/arborescens*, *Alt-a1* gene

GCCTCTCTCTTCGCCGCCGCTGGCCTTGCCGCTGCTGCTCCTCTCGAGTCTCGCCAGGAC

ACCGCATCCTGCCCTGTCACCACTGAGGGTGACTACGTCTGGAAGATCTCCGAATTCTAC

GGACGCAAGCCGGAAGGAACCTACTACAACAGCCTCGGCTTCAACATCAAGGCCACCAAC

GGAGGAACCCTCGACTTCACCTGCTCTGCTCAGGCCGATAAGCTTGAGGACCACAAGTGG

TACTCCTGTGGCGAGAACAGCTTCATGGACTTCTCTTTCGACAGCGACCGCAGCGGTCTG

CTCCTGAAGCAGAAGGTTAGCGACGAGTAAGTTACCCTTGTACCTTCGATTACTTCGCAG

ATTCAGATATACTAACATATTCCCAGCATCACCTATGTCGCTACCGCCACTCTTCCCAAC

TACTGCCGCGCTGGCGGTAACGGCCCTAAGGACTTTGTCTGCCAGGGTGTTGCCG

>A *Alternaria alternata/arborescens*, *Alt-a1* gene

CCTCTCTNTTCGCCGCCGCTGGCCTTGCCGCTGCTGCTCCTCTCGAGTCTCGCCAGGACA

CCGCATCCTGCCCTGTCACCACTGAGGGTGATTACGTCTGGAAGATCTCCGAATTCTACG

GACGCAAGCCGGAAGGAACCTACTACAACAGCCTCGGCTTCAACATCAAGGCCACCAACG

GAGGAACCCTCGACTTCACCTGCTCTGCTCAGGCCGATAAGCTTGAGGACCACAAGTGGT

ACTCCTGTGGCGAGAACAGCTTCATGGACTTCTCTTTCGACAGCGACCGCAGCGGTCTGC

TCCTGAAGCAGAAGGTTAGCGACGAGTAAGTTACCCTTGTACCTTCGATTACTTCGCAGA

TTCAGATATACTAACATATCCCCAGCATCACCTATGTCGCTACCGCCACTCTTCCCAACT

ACTGCCGCGCTGGCGGTAACGGCCCTAAGGACTTTGTCTGCCAGGGTGTTGCCG

>F12 *Alternaria alternata/arborescens*, *Alt-a1* gene

GCCTCTCTCTTCGCCGCCGCTGGCCTTGCCGCTGCTGCTCCTCTCGAGTCTCGCCAGGAC

ACCGCATCCTGCCCTGTCACCACTGAGGGTGACTACGTCTGGAAGATCTCCGAATTCTAC

GGACGCAAGCCGGAAGGAACCTACTACAACAGCCTCGGCTTCAACATCAAGGCCACCAAC

GGAGGAACCCTCGACTTCACCTGCTCTGCTCAGGCCGATAAGCTTGAGGACCACAAGTGG

TACTCCTGTGGCGAGAACAGCTTCATGGACTTCTCTTTCGACAGCGACCGCAGCGGTCTG

CTCCTGAAGCAGAAGGTTAGCGACGAGTAAGTTACCCTTGTACCTTCGATTACTTCGCAG

ATTCAGATATACTAACATATTCCCAGCATCACCTATGTCGCTACCGCCACTCTTCCCAAC

TACTGCCGCGCTGGCGGTAACGGCCCTAAGGACTTTGTCTGCCAGGGTGTTGCC

>F2C-F *Alternaria alternata/arborescens*, *Alt-a1* gene

CTCTCTTCGCCGCCGCTGGCCTTGCCGCTGCTGCTCCTCTCGAGTCTCGCCAGGACACCG

CATCCTGCCCTGTCACCACTGAGGGTGACTACGTCTGGAAGATCTCCGAATTCTACGGAC

GCAAGCCGGAAGGAACCTACTACAACAGCCTCGGCTTCAACATCAAGGCCACCAACGGAG

GAACCCTCGACTTCACCTGCTCTGCTCAGGCCGATAAGCTTGAGGACCACAAGTGGTACT

CCTGTGGCGAGAACAGCTTCATGGACTTCTCTTTCGACAGCGACCGCAGCGGTCTGCTCC

TGAAGCAGAAGGTTAGCGACGAGTAAGTTACCCTTGTACCTTCGATTACTTCGCAGATTC

AGATATACTAACATATTCCCAGCATCACCTATGTCGCTACCGCCACTCTTCCCAACTACT

GCCGCGCTGGCGGTAACGGCCCTAAGGACTTTGTCTGCCAGGGTGTTGCCGA

>F3-F *Alternaria alternata/arborescens*, *Alt-a1* gene

CGCCTCTCTCTTCGCCGCCGCTGGCCTTGCCGCTGCTGCTCCTCTCGAGTCTCGCCAGGA

CACCGCATCCTGCCCTGTCACCACTGAGGGTGACTACGTCTGGAAGATCTCCGAATTCTA

CGGACGCAAGCCGGAAGGAACCTACTACAACAGCCTCGGCTTCAACATCAAGGCCACCAA

CGGAGGAACCCTCGACTTCACCTGCTCTGCTCAGGCCGATAAGCTTGAGGACCACAAGTG

GTACTCCTGTGGCGAGAACAGCTTCATGGACTTCTCTTTCGACAGCGACCGCAGCGGTCT

GCTCCTGAAGCAGAAGGTTAGCGACGAGTAAGTTACCCTTGTACCTTCGATTACTTCGCA

GATTCAGATATACTAACATATTCCCAGCATCACCTATGTCGCTACCGCCACTCTTCCCAA

CTACTGCCGCGCTGGCGGTAACGGCCCTAAGGACTTTGTCTGCCAGGGTGTTGCCG

>F5C-F *Alternaria ovoidea*, *Alt-a1* gene

TCGCCTCTCTCTTCGCCGCCGCTGGCCTTCCCGCCGCTGCACCTCTCGAGTCTCGCCAGG

ACACCGCATCCTGCCCTGTCACCACCGAGGGTGACTACGTCTGGAAGATTTCCGAGTTCT

ACGGACGCAAGCCGGAGGGAACCTACTACAACAGCCTCGGCTTCAACATCAAGGCTACCA

ACGGAGGAACACTCGACTTCACCTGCTCTCACTCAGCCGACAAGCTTGAGGACCACACTT

GGTACTCTTGCGGCGAGAACAGCTTCATGGACTTCTCTTTCGACAGCGACCGCAACGGTC

TGCTCCTGAAGCAGAAGGTTAGCGACGAGTAAGTTACCCTTGTACCTTCGATTACTTCGC

AGATTCAGATATACTAACATGTTTCCAGCATCACCTATGTCGCTACCGCCACTCTTCCCA

ACTACTGCCGCGCTGGCGGTAACGGCCCTAAGGACTTTGTCTGCCAGGGTGTTGCC

>FC4 *Alternaria ovoidea*, *Alt-a1* gene

CCTCTCTCTTCGCCGCCGCTGGCCTTGCCGCCGCTGCACCTCTCGAGTCTCGCCAGGACA

CCGCATCCTGCCCTGTCACCACCGAGGGTGACTACGTCTGGAAGATTTCCGAGTTCTACG

GACGCAAGCCGGAGGGAACCTACTACAACAGCCTCGGCTTCAACATCAAGGCTACTAACG

GAGGAACACTCGACTTCACCTGCTCTCACTCAGCCGACAAGCTTGAGGACCACACTTGGT

ACTCTTGCGGCGAGAACAGCTTCATGGACTTCTCTTTCGACAGCGACCGCAACGGTCTGC

TCCTGAAGCAGAAGGTTAGCGACGAGTAAGTTACCCTTGTACCTTCGATTACTTCGCAGA

TTCAGATATACTAACATGTTTCCAGCATCACCTATGTCGCTACCGCCACTCTTCCCAACT

ACTGCCGCGCTGGCGGTAACGGCCCTAAGGACTTTGTCTGCCAGGG

>11C-F *Alternaria alternata/arborescens*, *gapdh* gene

AAGCGTAGTTNTGCCTAACTTGTCGATACAATNCTACCAGAGCTGACCGCATGCCACAGT

ATCGAGCACAACGACGTCGACATTGTCGCCGTAAACGACCCCTTCATCGAGCCCCACTAC

GCTGTAAGCTTCCCCAAGCACCCACACTACAGCCGCGGCCATCCAAGTTGCGAAAACAGT

CCTTGCGATGCGCTAGAGCTCCTCTGTGGTCGCAGAATGCAGGCTAACACATTCAGGCCT

ACATGCTCAAGTATGACAGCACACACGGCCAGTTCAAGGGTGAGATCAAGGTTGACGGCA

ACAACCTGACCGTCAACGGCAAGACCATCCGTTTCCACATGGAGAAGGACCCCGCCAACA

TCCCATGGAGCGAGACCGGCGCTTACTACGTCGTCGAGTCCACCGGTGTCTTCACCACCA

CCGAGAAGGCCAAGGCTCACTTGAAGGGTGGAGCCAAGAAGGTCGTCATTTCTGCTCCCT

CTGCTGACGCCCCCATGTTCGTTATGGGTGTCAACCACGAGACTTACAAGTCTGACATCG

AGGTTCTCTCCAACGCCTCTTG

>14C-F *Alternaria alternata/arborescens*, *gapdh* gene

CTTCYGSAATGCGTAAGTTTCGCCTAACTCGTCGATACAATCTACCAGAGCCTGACCGCA

TGCCACAGTATCGAGCACAACGACGTCGACATTGTCGCCGTAAACGACCCCTTCATCGAG

CCCCACTACGCTGTAAGCTTCCCCAAGCACCCACACTACAGCCGCGGCCATCCAAGTTGC

GAAAACAGTCCTTGCGATGCGCTAGAGCTCCTCTGTGGTCGCAGAATGCAGGCTAACACA

TTCAGGCCTACATGCTCAAGTATGACAGCACACACGGCCAGTTCAAGGGTGAGATCAAGG

TTGACGGCAACAACCTGACCGTCAACGGCAAGACCATCCGTTTCCACATGGAGAAGGACC

CCGCCAACATCCCATGGAGCGAGACCGGCGCTTACTACGTCGTCGAGTCCACCGGTGTCT

TCACCACCACCGAGAAGGCCAAGGCTCACTTGAAGGGTGGAGCCAAGAAGGTCGTCATTT

CTGCTCCCTCTGCTGACGCCCCCATGTTCGTTATGGGTGTCAACCACGAGACTTACAAGT

CTGACATCGAGGTTCTCTCCAACGCCTCTTGC

>15F *Alternaria alternata/arborescens*, *gapdh* gene

CGTATCGTYYTCCSMWAKYGYRWAAGTTTCGCCTAACTCGTCGATACAATCTACCAGAGC

TGACCGCATGCCACAGTATCGAGCACAACGACGTCGACATTGTCGCCGTAAACGACCCCT

TCATCGAGCCCCACTACGCTGTAAGCTTCCCCAAGCACCCACACTACAGCCGCGGCCATC

CAAGTTGCGAAAACAGTCCTTGCGATGCGCTAGAGCTCCTCTGTGGTCGCAGAATGCAGG

CTAACACATTCAGGCCTACATGCTCAAGTATGACAGCACACACGGCCAGTTCAAGGGTGA

GATCAAGGTTGACGGCAACAACCTGACCGTCAACGGCAAGACCATCCGTTTCCACATGGA

GAAGGACCCCGCCAACATCCCATGGAGCGAGACCGGCGCTTACTACGTCGTCGAGTCCAC

CGGTGTCTTCACCACCACCGAGAAGGCCAAGGCTCACTTGAAGGGTGGAGCCAAGAAGGT

CGTCATTTCTGCTCCCTCTGCTGACGCCCCCATGTTCGTTATGGGTGTCAACCACGAGAC

TTACAAGTCTGACATCGAGGTTCTCTCCAACGCCTCT

>1C-F *Alternaria ovoidea*, *gapdh* gene

ATTCGAANGCGTAAGGTTTTSSCCTAACTYGTCGATACAATNCTACCAGAGCTGACCGCA

TGCCACAGTATCGAGCACAACGACGTCGACATTGTCGCCGTAAACGACCCCTTCATCGAG

CCCCACTACGCTGTAAGCTTCCCCAAGCACCCACACTACAGCCGCGGCCATCCAAGTTGC

GAAAACAGTCCTTGCGATGCGCTAGAGCTCCTCTGTGGTCGCAGAATGCAGGCTAACACA

TTCAGGCCTACATGCTCAAGTATGACAGCACACACGGCCAGTTCAAGGGTGAGATCAAGG

TTGACGGCAACAACCTGACCGTCAACGGCAAGACCATCCGTTTCCACATGGAGAAGGACC

CCGCCAACATCCCATGGAGCGAGACCGGCGCTTACTACGTCGTCGAGTCCACCGGTGTCT

TCACCACCACCGAGAAGGCCAAGGCTCACTTGAAGGGTGGAGCCAAGAAGGTCGTCATTT

CTGCTCCCTCTGCTGACGCCCCCATGTTCGTTATGGGTGTCAACCACGAGACTTACAAGT

CTGACATCGAGGTTCTCTCCAACGCCTCTTGC

>2-F *Alternaria alternata/arborescens*, *gapdh* gene

GGCCGTATCGTCTTCYGSAATGCGTARGTTTCGCCTAACTCGTCGATACAATCTACCAGA

GCTGACCGCATGCCACAGTATCGAGCACAACGACGTCGACATTGTCGCCGTAAACGACCC

CTTCATCGAGCCCCACTACGCTGTAAGCTTCCCCAAGCACCCACACTACAGCCGCGGCCA

TCCAAGTTGCGAAAACAGTCCTTGCGATGCGCTAGAGCTCCTCTGTGGTCGCAGAATGCA

GGCTAACACATTCAGGCCTACATGCTCAAGTATGACAGCACACACGGCCAGTTCAAGGGT

GAGATCAAGGTTGACGGCAACAACCTGACCGTCAACGGCAAGACCATCCGTTTCCACATG

GAGAAGGACCCCGCCAACATCCCATGGAGCGAGACCGGCGCTTACTACGTCGTCGAGTCC

ACCGGTGTCTTCACCACCACCGAGAAGGCCAAGGCTCACTTGAAGGGTGGAGCCAAGAAG

GTCGTCATTTCTGCTCCCTCTGCTGACGCCCCCATGTTCGTTATGGGTGTCAACCACGAG

ACTTACAAGTCTGACATCGAGGTTCTCTCCAACGCCTCTT

>3-CF *Alternaria* *ovoidea*, *gapdh* gene

TCTNGAGCGTAGTTTCGCCTAACTCGTCGATACAATCTACCAGAGCTGACCGCATGCCAC

AGTATCGAGCACAACGACGTCGACATTGTCGCCGTAAACGACCCTTTCATCGAGCCCCAC

TACGCTGTAAGCTTCCCCAAGCACCCACACTACAGCCGCGGCCATCCAAGTTGCGAAAAC

AGTCCTTGCGATGCGCTAGAGCTCCTCTGTGGTCGCAGAATGCAGGCTAACACATTCAGG

CCTATATGCTCAAGTATGACAGCACACACGGCCAGTTCAAGGGTGAGATCAAGGTTGACG

GCAACAACCTGACCGTCAACGGCAAGACCATCCGTTTCCACATGGAGAAGGACCCCGCCA

ACATCCCATGGAGCGAGACCGGCGCTTACTACGTCGTCGAGTCCACCGGTGTCTTCACCA

CCACCGAGAAGGCCAAGGCTCACTTGAAGGGTGGAGCCAAGAAGGTCGTCATTTCTGCTC

CCTCTGCTGACGCCCCCATGTTCGTTATGGGTGTCAACCACGAGACTTACAAGTCTGACA

TCGAGGTTCTCTCCAACGCCTCTT

>4C-F *Alternaria alternata/arborescens*, *gapdh* gene

CCTCTTCAGCGTAGTTTCGCCTAACTCGTCGATACAATCTACCAGAGCTGACCGCATGCC

ACAGTATCGAGCACAACGACGTCGACATTGTCGCCGTAAACGACCCCTTCATCGAGCCCC

ACTACGCTGTAAGCTTCCCCAAGCACCCACACTACAGCCGCGGCCATCCAAGTTGCGAAA

ACAGTCCTTGCGATGCGCTAGAGCTCCTCTGTGGTCGCAGAATGCAGGCTAACACATTCA

GGCCTACATGCTCAAGTATGACAGCACACACGGCCAGTTCAAGGGTGAGATCAAGGTTGA

CGGCAACAACCTGACCGTCAACGGCAAGACCATCCGTTTCCACATGGAGAAGGACCCCGC

CAACATCCCATGGAGCGAGACCGGCGCTTACTACGTCGTCGAGTCCACCGGTGTCTTCAC

CACCACCGAGAAGGCCAAGGCTCACTTGAAGGGTGGAGCCAAGAAGGTCGTCATTTCTGC

TCCCTCTGCTGACGCCCCCATGTTCGTTATGGGTGTCAACCACGAGACTTACAAGTCTGA

CATCGAGGTTCTCTCCAACGCCTCTTGCAC

>5 *Alternaria alternata/arborescens*, *gapdh* gene

CNTCTNCCAAGCGTAGGTTTCGCCCTAACTCGTCGATACAATCTACCAGAGCTGACCGCA

TGCCACAGTATCGAGCACAACGACGTCGACATTGTCGCCGTAAACGACCCCTTCATCGAG

CCCCACTACGCTGTAAGCTTCCCCAAGCACCCACACTACAGCCGCGGCCATCCAAGTTGC

GAAAACAGTCCTTGCGATGCGCTAGAGCTCCTCTGTGGTCGCAGAATGCAGGCTAACACA

TTCAGGCCTACATGCTCAAGTATGACAGCACACACGGCCAGTTCAAGGGTGAGATCAAGG

TTGACGGCAACAACCTGACCGTCAACGGCAAGACCATCCGTTTCCACATGGAGAAGGACC

CCGCCAACATCCCATGGAGCGAGACCGGCGCTTACTACGTCGTCGAGTCCACCGGTGTCT

TCACCACCACCGAGAAGGCCAAGGCTCACTTGAAGGGTGGAGCCAAGAAGGTCGTCATTT

CTGCTCCCTCTGCTGACGCCCCCATGTTCGTTATGGGTGTCAACCACGAGACTTACAAGT

CTGACATCGAGGTTCTCTCCAACGCCTCTTGC

>5C *Alternaria ovoidea*, *gapdh* gene

TCTNGAAGCGTAGTTTCGCCCTAACTCGNTCGATACAATCTACCAGAGCTGACCGCATGC

CACAGTATCGAGCACAACGACGTCGACATTGTCGCCGTAAACGACCCCTTCATCGAGCCC

CACTACGCTGTAAGCTTCCCCAAGCACCCACACTACAGCCGCGGCCATCCAAGTTGCGAA

AACAGTCCTTGCGATGCGCTAGAGCTCCTCTGTGGTCGCAGAATGCAGGCTAACACATTC

AGGCCTACATGCTCAAGTATGACAGCACACACGGCCAGTTCAAGGGTGAGATCAAGGTTG

ACGGCAACAACCTGACCGTCAACGGCAAGACCATCCGTTTCCACATGGAGAAGGACCCCG

CCAACATCCCATGGAGCGAGACCGGCGCTTACTACGTCGTCGAGTCCACCGGTGTCTTCA

CCACCACCGAGAAGGCCAAGGCTCACTTGAAGGGTGGAGCCAAGAAGGTCGTCATTTCTG

CTCCCTCTGCTGACGCCCCCATGTTCGTTATGGGTGTCAACCACGAGACTTACAAGTCTG

ACATCGAGGTTCTCTCCAACGCCTCTT

>6C *Alternaria ovoidea*, *gapdh* gene

GCCGTATNGTNTTCCGCRATGCGTAAGTTTCGCCTAACTCGTCGATACAATCTACCAGAG

CTGACCGCATGCCACAGTATCGAGCACAACGACGTCGACATTGTCGCCGTAAACGACCCC

TTCATCGAGCCCCACTACGCTGTAAGCTTCCCCAAGCACCCACACTACAGCCGCGGCCAT

CCAAGTTGCGAAAACAGTCCTTGCGATGCGCTAGAGCTCCTCTGTGGTCGCAGAATGCAG

GCTAACACATTCAGGCCTACATGCTCAAGTATGACAGCACACACGGCCAGTTCAAGGGTG

AGATCAAGGTTGACGGCAACAACCTGACCGTCAACGGCAAGACCATCCGTTTCCACATGG

AGAAGGACCCCGCCAACATCCCATGGAGCGAGACCGGCGCTTACTACGTCGTCGAGTCCA

CCGGTGTCTTCACCACCACCGAGAAGGCCAAGGCTCACTTGAAGGGTGGAGCCAAGAAGG

TCGTCATTTCTGCTCCCTCTGCTGACGCCCCCATGTTCGTTATGGGTGTCAACCACGAGA

CTTACAAGTCTGACATCGAGGTTCTCTCCAACGCCTCTTGC

>7 *Alternaria alternata/arborescens*, *gapdh* gene

GNATTCGAAGGCGTAAGTTNTGCCCTAACTTGKTCGATACAATNCTACCAGAGCTGACCG

CATGCCACAGTATCGAGCACAACGACGTCGACATTGTCGCCGTAAACGACCCCTTCATCG

AGCCCCACTACGCTGTAAGCTTCCCCAAGCACCCACACTACAGCCGCGGCCATCCAAGTT

GCGAAAACAGTCCTTGCGATGCGCTAGAGCTCCTCTGTGGTCGCAGAATGCAGGCTAACA

CATTCAGGCCTACATGCTCAAGTATGACAGCACACACGGCCAGTTCAAGGGTGAGATCAA

GGTTGACGGCAACAACCTGACCGTCAACGGCAAGACCATCCGTTTCCACATGGAGAAGGA

CCCCGCCAACATCCCATGGAGCGAGACCGGCGCTTACTACGTCGTCGAGTCCACCGGTGT

CTTCACCACCACCGAGAAGGCCAAGGCTCACTTGAAGGGTGGAGCCAAGAAGGTCGTCAT

TTCTGCTCCCTCTGCTGACGCCCCCATGTTCGTTATGGGTGTCAACCACGAGACTTACAA

GTCTGACATCGAGGTTCTCTCCAACG

>7C *Alternaria* species, *gapdh* gene

CGTTCYKYAATGCGTAAGTTWYGCNTAACTCGTCGATACAATACTACCAGAGCTGACCGC

ATGCCACAGTATCGAGCACAACGACGTCGACATTGTCGCCGTAAACGACCCCTTCATCGA

GCCCCACTACGCTGTAAGCTTCCCCAAGCACCCACACTACAGCCGCGGCCATCCAAGTTG

CGAAAACAGTCCTTGCGATGCGCTAGAGCTCCTCTGTGGTCGCAGAATGCAGGCTAACAC

ATTCAGGCCTACATGCTCAAGTATGACAGCACACACGGCCAGTTCAAGGGTGAGATCAAG

GTTGACGGCAACAACCTGACCGTCAACGGCAAGACCATCCGTTTCCACATGGAGAAGGAC

CCCGCCAACATCCCATGGAGCGAGACCGGCGCTTACTACGTCGTCGAGTCCACCGGTGTC

TTCACCACCACCGAGAAGGCCAAGGCTCACTTGAAGGGTGGAGCCAAGAAGGTCGTCATT

TCTGCTCCCTCTGCTGACGCCCCCATGTTCGTTATGGGTGTCAACCACGAGACTTACAAG

TCTGACATCGAGGTTCTCT

>8 *Alternaria alternata/arborescens*, *gapdh* gene

CGTNTTCAGCGTTAGTTTGCCTAACTCGTTCGATACAATCTACCAGAGCTGACCGCATGC

CACAGTATCGAGCACAACGACGTCGACATTGTCGCCGTAAACGACCCCTTCATCGAGCCC

CACTACGCTGTAAGCTTCCCCAAGCACCCACACTACAGCCGCGGCCATCCAAGTTGCGAA

AACAGTCCTTGCGATGCGCTAGAGCTCCTCTGTGGTCGCAGAATGCAGGCTAACACATTC

AGGCCTACATGCTCAAGTATGACAGCACACACGGCCAGTTCAAGGGTGAGATCAAGGTTG

ACGGCAACAACCTGACCGTCAACGGCAAGACCATCCGTTTCCACATGGAGAAGGACCCCG

CCAACATCCCATGGAGCGAGACCGGCGCTTACTACGTCGTCGAGTCCACCGGTGTCTTCA

CCACCACCGAGAAGGCCAAGGCTCACTTGAAGGGTGGAGCCAAGAAGGTCGTCATTTCTG

CTCCCTCTGCTGACGCCCCCATGTTCGTTATGGGTGTCAACCACGAGACTTACAAGTCTG

ACATCGAGGTTCTCTCCAACGCC

>8-C *Alternaria alternata/arborescens*, *gapdh* gene

TCTNCNAGCGTAGGTTTCGCCNTAACTCGTCGATACAATCTACCAGAGCTGACCGCATGC

CACAGTATCGAGCACAACGACGTCGACATTGTCGCCGTAAACGACCCCTTCATCGAGCCC

CACTACGCTGTAAGCTTCCCCAAGCACCCACACTACAGCCGCGGCCATCCAAGTTGCGAA

AACAGTCCTTGCGATGCGCTAGAGCTCCTCTGTGGTCGCAGAATGCAGGCTAACACATTC

AGGCCTACATGCTCAAGTATGACAGCACACACGGCCAGTTCAAGGGTGAGATCAAGGTTG

ACGGCAACAACCTGACCGTCAACGGCAAGACCATCCGTTTCCACATGGAGAAGGACCCCG

CCAACATCCCATGGAGCGAGACCGGCGCTTACTACGTCGTCGAGTCCACCGGTGTCTTCA

CCACCACCGAGAAGGCCAAGGCTCACTTGAAGGGTGGAGCCAAGAAGGTCGTCATTTCTG

CTCCCTCTGCTGACGCCCCCATGTTCGTTATGGGTGTCAACCACGAGACTTACAAGTCTG

ACATCGAGGTTCTCTCCAACGCCTCTTG

>9-F *Alternaria alternata/arborescens*, *gapdh* gene

CGNTTNGAAGCGTAGTTWYGCCTAACTYGTCGATACAATCTACCAGAGCTGACCGCATGC

CACAGTATCGAGCACAACGACGTCGACATTGTCGCCGTAAACGACCCCTTCATCGAGCCC

CACTACGCTGTAAGCTTCCCCAAGCACCCACACTACAGCCGCGGCCATCCAAGTTGCGAA

AACAGTCCTTGCGATGCGCTAGAGCTCCTCTGTGGTCGCAGAATGCAGGCTAACACATTC

AGGCCTACATGCTCAAGTATGACAGCACACACGGCCAGTTCAAGGGTGAGATCAAGGTTG

ACGGCAACAACCTGACCGTCAACGGCAAGACCATCCGTTTCCACATGGAGAAGGACCCCG

CCAACATCCCATGGAGCGAGACCGGCGCTTACTACGTCGTCGAGTCCACCGGTGTCTTCA

CCACCACCGAGAAGGCCAAGGCTCACTTGAAGGGTGGAGCCAAGAAGGTCGTCATTTCTG

CTCCCTCTGCTGACGCCCCCATGTTCGTTATGGGTGTCAACCACGAGACTTACAAGTCTG

ACATCGAGGTTCTCTCCAACGC

>A *Alternaria alternata/arborescens*, *gapdh* gene

TCTNNAGCGTAGTTTCGCCTAACTCGTCGATACAATCTACCAGAGCTGACCGCATGCCAC

AGTATCGAGCACAACGACGTCGACATTGTCGCCGTAAACGACCCCTTCATCGAGCCCCAC

TACGCTGTAAGCTTCCCCAAGCACCCACACTACAGCCGCGGCCATCCAAGTTGCGAAAAC

AGTCCTTGCGATGCGCTAGAGCTCCTCTGTGGTCGCAGAATGCAGGCTAACACATTCAGG

CCTACATGCTCAAGTATGACAGCACACACGGCCAGTTCAAGGGTGAGATCAAGGTTGACG

GCAACAACCTGACCGTCAACGGCAAGACCATCCGTTTCCACATGGAGAAGGACCCCGCCA

ACATCCCATGGAGCGAGACCGGCGCTTACTACGTCGTCGAGTCCACCGGTGTCTTCACCA

CCACCGAGAAGGCCAAGGCTCACTTGAAGGGTGGAGCCAAGAAGGTCGTCATTTCTGCTC

CCTCTGCTGACGCCCCCATGTTCGTTATGGGTGTCAACCACGAGACTTACAAGTCTGACA

TCGAGGTTCTCTCCAACGCCTCTTG

>F12 *Alternaria alternata/arborescens*, *gapdh* gene

GGCCGTATCGTCTTCYGCMATGCGTARKTTTCGCCTAACTCGTCGATACAATCTACCAGA

GCTGACCGCATGCCACAGTATCGAGCACAACGACGTCGACATTGTCGCCGTAAACGACCC

CTTCATCGAGCCCCACTACGCTGTAAGCTTCCCCAAGCACCCACACTACAGCCGCGGCCA

TCCAAGTTGCGAAAACAGTCCTTGCGATGCGCTAGAGCTCCTCTGTGGTCGCAGAATGCA

GGCTAACACATTCAGGCCTACATGCTCAAGTATGACAGCACACACGGCCAGTTCAAGGGT

GAGATCAAGGTTGACGGCAACAACCTGACCGTCAACGGCAAGACCATCCGTTTCCACATG

GAGAAGGACCCCGCCAACATCCCATGGAGCGAGACCGGCGCTTACTACGTCGTCGAGTCC

ACCGGTGTCTTCACCACCACCGAGAAGGCCAAGGCTCACTTGAAGGGTGGAGCCAAGAAG

GTCGTCATTTCTGCTCCCTCTGCTGACGCCCCCATGTTCGTTATGGGTGTCAACCACGAG

ACTTACAAGTCTGACATCGAGGTTCTCTCCAACGCCTCT

>F3-F *Alternaria alternata/arborescens*, *gapdh* gene

TCTNGCAGCGTAGTTTCGCCNTAACTCGTCGATACAATCTACCAGAGCTGACCGCATGCC

ACAGTATCGAGCACAACGACGTCGACATTGTCGCCGTAAACGACCCCTTCATCGAGCCCC

ACTACGCTGTAAGCTTCCCCAAGCACCCACACTACAGCCGCGGCCATCCAAGTTGCGAAA

ACAGTCCTTGCGATGCGCTAGAGCTCCTCTGTGGTCGCAGAATGCAGGCTAACACATTCA

GGCCTACATGCTCAAGTATGACAGCACACACGGCCAGTTCAAGGGTGAGATCAAGGTTGA

CGGCAACAACCTGACCGTCAACGGCAAGACCATCCGTTTCCACATGGAGAAGGACCCCGC

CAACATCCCATGGAGCGAGACCGGCGCTTACTACGTCGTCGAGTCCACCGGTGTCTTCAC

CACCACCGAGAAGGCCAAGGCTCACTTGAAGGGTGGAGCCAAGAAGGTCGTCATTTCTGC

TCCCTCTGCTGACGCCCCCATGTTCGTTATGGGTGTCAACCACGAGACTTACAAGTCTGA

CATCGAGGTTCTCTCCAACGCCTCTT

>F5C-F *Alternaria ovoidea*, *gapdh* gene

GTCTNGNAGCGTAGTTTCGCCNTAACTCGTCGATACAATCTACCAGAGCTGACCGCATGC

CACAGTATCGAGCACAACGACGTCGACATTGTCGCCGTAAACGACCCCTTCATCGAGCCC

CACTACGCTGTAAGCTTCCCCAAGCACCCACACTACAGCCGCGGCCATCCAAGTTGCGAA

AACAGTCCTTGCGATGCGCTAGAGCTCCTCTGTGGTCGCAGAATGCAGGCTAACACATTC

AGGCCTACATGCTCAAGTATGACAGCACACACGGCCAGTTCAAGGGTGAGATCAAGGTTG

ACGGCAACAACCTGACCGTCAACGGCAAGACCATCCGTTTCCACATGGAGAAGGACCCCG

CCAACATCCCATGGAGCGAGACCGGCGCTTACTACGTCGTCGAGTCCACCGGTGTCTTCA

CCACCACCGAGAAGGCCAAGGCTCACTTGAAGGGTGGAGCCAAGAAGGTCGTCATTTCTG

CTCCCTCTGCTGACGCCCCCATGTTCGTTATGGGTGTCAACCACGAGACTTACAAGTCTG

ACATCGAGGTTCTCTCCAACGCCTCTTGCA

>FC4 *Alternaria ovoidea*, *gapdh* gene

CGTNTTGAAGCGTNGTTTGCCTAACTCGTCGATACAATCTACCAGAGCTGACCGCATGCC

ACAGTATCGAGCACAACGACGTCGACATTGTCGCCGTAAACGACCCCTTCATCGAGCCCC

ACTACGCTGTAAGCTTCCCCAAGCACCCACACTACAGCCGCGGCCATCCAAGTTGCGAAA

ACAGTCCTTGCGATGCGCTAGAGCTCCTCTGTGGTCGCAGAATGCAGGCTAACACATTCA

GGCCTACATGCTCAAGTATGACAGCACACACGGCCAGTTCAAGGGTGAGATCAAGGTTGA

CGGCAACAACCTGACCGTCAACGGCAAGACCATCCGTTTCCACATGGAGAAGGACCCCGC

CAACATCCCATGGAGCGAGACCGGCGCTTACTACGTCGTCGAGTCCACCGGTGTCTTCAC

CACCACCGAGAAGGCCAAGGCTCACTTGAAGGGTGGAGCCAAGAAGGTCGTCATTTCTGC

TCCCTCTGCTGACGCCCCCATGTTCGTTATGGGTGTCAACCACGAGACTTACAAGTCTGA

CATCGAGGTTCTCTCC

>6 *Alternaria alternata/arborescens*, *gapdh* gene

GCCGTATCGTCTTCCKCTARWGCGTAAGTTTCGCCTAACTCGTCGATACAATCTACCAGA

GCTGACCGCATGCCACAGTATCGAGCACAACGACGTCGACATTGTCGCCGTAAACGACCC

CTTCATCGAGCCCCACTACGCTGTAAGCTTCCCCAAGCACCCACACTACAGCCGCGGCCA

TCCAAGTTGCGAAAACAGTCCTTGCGATGCGCTAGAGCTCCTCTGTGGTCGCAGAATGCA

GGCTAACACATTCAGGCCTACATGCTCAAGTATGACAGCACACACGGCCAGTTCAAGGGT

GAGATCAAGGTTGACGGCAACAACCTGACCGTCAACGGCAAGACCATCCGTTTCCACATG

GAGAAGGACCCCGCCAACATCCCATGGAGCGAGACCGGCGCTTACTACGTCGTCGAGTCC

ACCGGTGTCTTCACCACCACCGAGAAGGCCAAGGCTCACTTGAAGGGTGGAGCCAAGAAG

GTCGTCATTTCTGCTCCCTCTGCTGACGCCCCCATGTTCGTTATGGGTGTCAACCACGAG

ACTTACAAGTCTGACATCGAGGTTCTCTCCAACGCCTCTTGC

>F2C-F *Alternaria alternata/arborescens*, *gapdh* gene

TCCKCTWRWGCGTAAGTTTCGCCTAACTCGTCGATACAATCTACCAGAGCTGACCGCATG

CCACAGTATCGAGCACAACGACGTCGACATTGTCGCCGTAAACGACCCCTTCATCGAGCC

CCACTACGCTGTAAGCTTCCCCAAGCACCCACACTACAGCCGCGGCCATCCAAGTTGCGA

AAACAGTCCTTGCGATGCGCTAGAGCTCCTCTGTGGTCGCAGAATGCAGGCTAACACATT

CAGGCCTACATGCTCAAGTATGACAGCACACACGGCCAGTTCAAGGGTGAGATCAAGGTT

GACGGCAACAACCTGACCGTCAACGGCAAGACCATCCGTTTCCACATGGAGAAGGACCCC

GCCAACATCCCATGGAGCGAGACCGGCGCTTACTACGTCGTCGAGTCCACCGGTGTCTTC

ACCACCACCGAGAAGGCCAAGGCTCACTTGAAGGGTGGAGCCAAGAAGGTCGTCATTTCT

GCTCCCTCTGCTGACGCCCCCATGTTCGTTATGGGTGTCAACCACGAGACTTACAAGTCT

GACATCGAGGTTCNTCTCC

>A *Alternaria alternata/arborescens*, *rpb2* gene

GGTAATGAATTCACCGCCTTTTGCAGTGGTGTGCTGGGTCGTCGCGACGAGGACGATCGT

GATCACTTCGGTAAGAAGCGTCTGGATCTTGCAGGACCCTTGGTCGCCAACTTGTTCCGT

ATCCTCTTCCTGAAGCTCACCAAGGACGTATACAAGTACCTCCAGCGATGTGTTGAGAAC

AACCAGGATTTCAACGTTCAGATGGCTGTCAAGGCCAGCATCATCACAAATGGCCTGAAA

TACTCCCTGGCAACAGGAAACTGGGGTGACCAGAAGAAGGCCGCGTCCGCGAAAGCCGGT

GTCTCTCAGGTGTTGAACCGATACACTTACGCCTCCACACTGTCCCATTTGCGTCGAACG

AATACCCCTGTTGGTCGTGATGGAAAGCTGGCCAAGCCGCGACAACTCCACAACTCTCAT

TGGGGTCTTGTCTGCCCTGCCGAGACTCCTGAAGGACAGGCTTGTGGTTTGGTTAAGAAC

CTGTCTTTGATGTGCTATGTTAGTGTCGGTAGTGACGCTTCTCCCATCATCGACTTCATG

ACACAACGAAACATGCAACTTCTAGAAGAGTATGATCAGAACCAGAACCCCGATGCCACC

AAGGTCTTCGTCAATGGTGTCTGGGTTGGTGTCCATTCCAACGCTCAACAACTTGTCACA

GTTGTGCAGGAGCTGCGACGAAACGGAACTCTATCCTATGAGATGAGTCTGATTCGTGAC

ATTCGTGACCGAGAGTTCAAGATCTTCACAGATGCTGGCCGTGTCATGAGACCACTGTTC

GTTGTTGAGAACGACATTCGGAAGCCAAACCGCAACCACCTCATCTTCACCAAGGAGATC

AGTAACAAGCTCAAGCAGGAGCAACAAGAGACAAGCACACGACAGGGTTGGAGCCAGGAT

GAGGTCGAATCAGCTACCTACGGCTGGAGAGGTCTTATTCAAGACGGTGTTGTGGAGTAC

CTAGACGCTGAAGAGGAGGAGACCGCCATGATAACGTTCTCCCCTGAGGATCTGGAGGAG

TGGCGAGAGATGAAGTTGGGCTTGCCGGCGGCGGAGCGATCCACCGAAGGTGAGCACCGT

CTCCGACGCCTCAAGCCACTACCAGACCCCCGCATCCATGCCTATACACATTGCGAGATT

CATCCGGCTATGATTCTCGGTATCTGTGCCCAGTATCA

>11C-F *Alternaria alternata/arborescens*, *rpb2* gene

TGGANTTGCTGGACCNTGGGTCGCCAACTTTGTTTCCGTATCCTCTTCCTGAAGCTCACC

AAGGACGTATACAAGTACCTCCAGCGATGTGTTGAGAACAACCAGGATTTCAACGTTCAG

ATGGCTGTCAAGGCCAGCATCATCACAAACGGCCTGAAATACTCCCTGGCAACAGGAAAC

TGGGGTGACCAGAAGAAGGCCGCTTCCGCGAAAGCCGGTGTCTCCCAGGTGTTGAACCGA

TACACTTACGCCTCCACACTGTCCCATTTGCGTCGAACGAATACCCCTGTTGGTCGTGAT

GGAAAGCTGGCCAAGCCGCGACAACTCCACAACTCTCATTGGGGTCTTGTCTGCCCTGCC

GAGACTCCTGAAGGACAGGCTTGTGGTCTGGTTAAGAACCTGTCTTTGATGTGCTATGTT

AGTGTCGGTAGTGACGCTTCTCCCATCATCGACTTCATGACACAACGAAACATGCAACTT

CTAGAAGAGTATGATCAGAACCAGAACCCCGATGCCACCAAGGTCTTCGTCAACGGTGTC

TGGGTTGGTGTCCATTCCAACGCTCAACAACTTGTCACAGTTGTGCAGGAGCTGCGACGA

AACGGAACTCTATCCTATGAGATGAGTCTGATTCGTGACATTCGTGACCGAGAGTTCAAG

ATCTTCACAGATGCTGGCCGTGTCATGAGACCACTGTTCGTTGTTGAGAACGACATTCGG

AAGCCAAACCGCAACCACCTCATCTTCACCAAGGAGATCAGTAACAAGCTCAAGCAGGAG

CAACAAGAGACAAGCACACGACAGGGTTGGAGCCAGGATGAGGTCGAATCAGCTACCTAC

GGCTGGAGAGGTCTCATTCAAGACGGTGTTGTGGAGTACCTTGACGCTGAAGAGGAGGAG

ACCGCCATGATAACGTTCTCCCCTGAGGATCTGGAGGAGTGGCGAGAGATGAAATTGGGC

TTGCCGGCGGCGGAGCGATCCACCGAGGGTGAGCACCGTCTCCGACGCCTCAAGCCACTA

CCAGACCCCCGCATCCATGCCTATATCTCATTGCGAGATTCNTAAAGGGTATGATTCTCG

GTATCTGTGCCAGTATCATCCCTTCCCCGATCACACCATCGCCCTACTTTT

>14C-F *Alternaria alternata/arborescens*, *rpb2* gene

ATGGANTGCNGGACCTTGGTCGCCAACTTGTTTCCGTATCCTCTTCCTGAAGCTCACCAA

GGATGTGNTACAATNTACCTTCCAGCGATGTGTTGAGAACAACCAGGATTTCAACGTTCA

GATGGCTGTCAAGGCCAGCATCATCACAAACGGCCTGAAATACTCCCTGGCAACAGGAAA

CTGGGGTGACCAGAAGAAGGCCGCTTCCGCGAAAGCCGGTGTCTCCCAGGTGTTGAACCG

ATACACTTACGCCTCCACACTGTCACATTTGCGTCGAACGAATACCCCTGTTGGTCGTGA

TGGAAAGCTGGCCAAGCCGCGACAACTCCACAACTCTCATTGGGGTCTTGTCTGCCCTGC

CGAGACTCCTGAAGGACAGGCTTGTGGTCTGGTTAAGAACCTGTCTTTGATGTGCTATGT

TAGTGTCGGTAGTGACGCTTCTCCCATCATCGACTTCATGACACAACGAAACATGCAACT

CCTAGAAGAGTATGATCAGAACCAGAACCCCGATGCCACCAAGGTCTTTGTCAACGGTGT

CTGGGTTGGTGTCCATTCCAACGCTCAACAACTTGTCACAGTTGTGCAGGAGCTGCGACG

AAACGGAACTCTATCCTATGAGATGAGTCTGATTCGTGACATTCGTGACCGAGAGTTCAA

GATCTTCACAGATGCTGGCCGTGTCATGAGACCACTGTTCGTTGTTGAGAACGACATTCG

GAAGCCAAACCGCAACCACCTCATCTTCACCAAGGAGATCAGTAACAAGCTCAAGCAGGA

ACAACAAGAGACAAGCACACGACAGGGTTGGAGCCAGGATGAGGTCGAATCAGCTACCTA

CGGCTGGAGAGGTCTTATTCAAGACGGTGTTGTGGAGTACCTAGACGCTGAAGAGGAGGA

GACCGCCATGATAACGTTCTCCCCTGAGGATCTGGAGGAGTGGCGAGAGATGAAATTGGG

TTTGCCGGCGGCGGAGCGATCCACCGAGGGTGAGCACCGTCTCCGACGCCTCAAGCCACT

ACCAGACCCCCGCATCCATGCCTATACTCATTGCGAGATTCATCCGGCTATGATTCTCGG

TATCTGTGCCAGTATCATTCCCTCCCCGATCACAACCATCGNCCCTAACNTTT

>15F *Alternaria alternata/arborescens*, *rpb2* gene

TGGANTGCTGGACCCTTGGTCGCCACTTGTTCCGTATCCTCTTCCTGAAGCTCACCAAGG

ACGTATACAAGTTACCTCCAGCGAATGTTGTTGAGAACAACCAGGATTTCAACGTTCAGA

TGGCTGTCAAGGCCAGCATCATCACAAACGGCCTGAAATACTCCCTGGCAACAGGAAACT

GGGGTGACCAGAAGAAGGCCGCTTCCGCGAAAGCCGGTGTCTCCCAGGTGTTGAACCGAT

ACACTTACGCCTCCACACTGTCCCATTTGCGTCGAACGAATACCCCTGTTGGTCGTGATG

GAAAGCTGGCCAAGCCGCGACAACTCCACAACTCTCATTGGGGTCTTGTCTGCCCTGCCG

AGACTCCTGAAGGACAGGCTTGTGGTCTGGTTAAGAACCTGTCTTTGATGTGCTATGTTA

GTGTCGGTAGTGACGCTTCTCCCATCATCGACTTCATGACACAACGAAACATGCAACTTC

TAGAAGAGTATGATCAGAACCAGAACCCCGATGCCACCAAGGTCTTCGTCAACGGTGTCT

GGGTTGGTGTCCATTCCAACGCTCAACAACTTGTCACAGTTGTGCAGGAGCTGCGACGAA

ACGGAACTCTATCCTATGAGATGAGTCTGATTCGTGACATTCGTGACCGAGAGTTCAAGA

TCTTCACAGATGCTGGCCGTGTCATGAGACCACTGTTCGTTGTTGAGAACGACATTCGGA

AGCCAAACCGCAACCACCTCATCTTCACCAAGGAGATCAGTAACAAGCTCAAGCAGGAGC

AACAAGAGACAAGCACACGACAGGGTTGGAGCCAGGATGAGGTCGAATCAGCTACCTACG

GCTGGAGAGGTCTCATTCAAGACGGTGTTGTGGAGTACCTTGACGCTGAAGAGGAGGAGA

CCGCCATGATAACGTTCTCCCCTGAGGATCTGGAGGAGTGGCGAGAGATGAAATTGGGCT

TGCCGGCGGCGGAGCGATCCACCGAGGGTGAGCACCGTCTCCGACGCCTCAAGCCACTAC

CAGACCCCCGCATCCATGCCTATACTCATTGCGAGATTCATCCGGCTATGATTCTCGGTA

TCTGTGCCAGTATCATTCCCTCCCCGATCACAACCATCGCCCCNACCTTT

>1C-F *Alternaria ovoidea*, *rpb2* gene

TGGATTGCGGACCCTTGGTCGCCACTTGGTTCCGTATCCTCTTCCTGAAGCTCACCAAGG

ATGTGNCCNNTTTACCTCCAGCGAATGTGTTTGAGAACAACCAGGATTTCAACGTTCAGA

TGGCTGTCAAGGCCAGCATCATCACAAACGGCCTGAAATACTCCCTGGCAACAGGAAACT

GGGGTGACCAGAAGAAGGCCGCTTCCGCGAAAGCCGGTGTCTCCCAGGTGTTGAACCGAT

ACACTTACGCCTCCACACTGTCACATTTGCGTCGAACGAATACCCCTGTTGGTCGTGATG

GAAAGCTGGCCAAGCCGCGACAACTCCACAACTCTCATTGGGGTCTTGTCTGCCCTGCCG

AGACTCCTGAAGGACAGGCTTGTGGTCTGGTTAAGAACCTGTCTTTGATGTGCTATGTTA

GTGTCGGTAGTGACGCTTCTCCCATCATCGACTTCATGACACAACGAAACATGCAACTCC

TAGAAGAGTATGATCAGAACCAGAACCCCGATGCCACCAAGGTCTTTGTCAACGGTGTCT

GGGTTGGTGTCCATTCCAACGCTCAACAACTTGTCACAGTTGTGCAGGAGCTGCGACGAA

ACGGAACTCTATCCTATGAGATGAGTCTGATTCGTGACATTCGTGACCGAGAGTTCAAGA

TCTTCACAGATGCTGGCCGTGTCATGAGACCACTGTTCGTTGTTGAGAACGACATTCGGA

AGCCAAACCGCAACCACCTCATCTTCACCAAGGAGATCAGTAACAAGCTCAAGCAGGAAC

AACAAGAGACAAGCACACGACAGGGTTGGAGCCAGGATGAGGTCGAATCAGCTACCTACG

GCTGGAGAGGTCTTATTCAAGACGGTGTTGTGGAGTACCTAGACGCTGAAGAGGAGGAGA

CCGCCATGATAACGTTCTCCCCTGAGGATCTGGAGGAGTGGCGAGAGATGAAATTGGGTT

TGCCGGCGGCGGAGCGATCCACCGAGGGTGAGCACCGTCTCCGACGCCTCAAGCCACTAC

CAGACCCCCGCATCCATGCCTATACTCATTGCGAGATTCATCCGGCTATGATTCTCGGTA

TCTGTGCCAGTATCATTCCCTCCCCGATCACAACCATCGCCCCTAACCTTT

>2-F *Alternaria alternata/arborescens*, *rpb2* gene

TGGATTGCGGACCTTGGTCGCCACTTGTTCCGTATCCTCTTCCTGAAGCTCACCAAGGAC

GTATACAAGTTACCTCCAGCGATGTGTTGAGAACAACCAGGATTTCAACGTTCAGATGGC

TGTCAAGGCCAGCATCATCACAAATGGCCTGAAATACTCCCTGGCAACAGGAAACTGGGG

TGACCAGAAGAAGGCCGCGTCCGCGAAAGCCGGTGTCTCTCAGGTGTTGAACCGATACAC

TTACGCCTCCACACTGTCCCATTTGCGTCGAACGAATACCCCTGTTGGTCGTGATGGAAA

GCTGGCCAAGCCGCGACAACTCCACAACTCTCATTGGGGTCTTGTCTGCCCTGCCGAGAC

TCCTGAAGGACAGGCTTGTGGTTTGGTTAAGAACCTGTCTTTGATGTGCTATGTTAGTGT

CGGTAGTGACGCTTCTCCCATCATCGACTTCATGACACAACGAAACATGCAACTTCTAGA

AGAGTATGATCAGAACCAGAACCCCGATGCCACCAAGGTCTTCGTCAATGGTGTCTGGGT

TGGTGTCCATTCCAACGCTCAACAACTTGTCACAGTTGTGCAGGAGCTGCGACGAAACGG

AACTCTATCCTATGAGATGAGTCTGATTCGTGACATTCGTGACCGAGAGTTCAAGATCTT

CACAGATGCTGGCCGTGTCATGAGACCACTGTTCGTTGTTGAGAACGACATTCGGAAGCC

AAACCGCAACCACCTCATCTTCACCAAGGAGATCAGTAACAAGCTCAAGCAGGAGCAACA

AGAGACAAGCACACGACAGGGTTGGAGCCAGGATGAGGTCGAATCAGCTACCTACGGCTG

GAGAGGTCTTATTCAAGACGGTGTTGTGGAGTACCTAGACGCTGAAGAGGAGGAGACCGC

CATGATAACGTTCTCCCCTGAGGATCTGGAGGAGTGGCGAGAGATGAAGTTGGGCTTGCC

GGCGGCGGAGCGATCCACCGAAGGTGAGCACCGTCTCCGACGCCTCAAGCCACTACMAGA

CCCCCGCATCCATGCCTATACACATTGCGAGATTCATNAGGNTATGATTCTCGGTATCTG

TGCCAGTATCATTCCCTCCCCGATCACAACCATCGCCCNAACCTTTCC

>3-CF *Alternaria ovoidea*, *rpb2* gene

ANATTGGATTGCGGACCNTTGGTCGCCACTTGTTCCGTATCCTCTTCCTGAAGCTCACCA

AGGACGTATACAAGTACCTCCAGCGATGTGTTGAGAACAACCAGGATTTCAACGTTCAGA

TGGCTGTCAAGGCCAGCATCATCACAAACGGCCTGAAATACTCACTGGCAACAGGAAACT

GGGGTGACCAGAAGAAGGCCGCTTCCGCGAAAGCCGGTGTCTCCCAGGTGTTGAACCGAT

ACACTTACGCCTCCACACTGTCCCATTTGCGTCGAACGAATACCCCTGTTGGTCGTGACG

GAAAGCTGGCCAAGCCGCGACAACTCCACAACTCTCATTGGGGTCTCGTCTGCCCTGCTG

AGACTCCTGAAGGACAGGCTTGTGGTCTGGTTAAGAACCTGTCTTTGATGTGCTATGTTA

GTGTCGGTAGTGACGCTTCTCCCATCATCGACTTCATGACACAACGAAATATGCAACTTC

TAGAAGAGTATGATCAGAACCAGAACCCCGATGCCACCAAGGTCTTCGTCAACGGTGTCT

GGGTTGGTGTCCATTCCAACGCTCAACAACTTGTCACAGTTGTGCAGGAGCTGCGACGAA

ACGGAACTCTATCCTATGAGATGAGTCTGATTCGTGACATTCGTGACCGAGAGTTCAAGA

TCTTCACAGATGCTGGCCGTGTCATGAGACCGCTGTTCGTTGTTGAGAACGACATTCGGA

AGCCAAACCGCAACCACCTCATCTTCACCAAGGAGATCAGTAACAAGCTCAAGCAGGAGC

AACAAGAGACAAGCACACGCCAGGGTTGGAGCCAGGATGAGGTCGAATCAGCTACCTACG

GCTGGAGAGGTCTTATTCAAGACGGTGTTGTGGAGTACCTAGACGCTGAAGAGGAGGAGA

CCGCCATGATAACGTTCTCCCCTGAGGATCTGGAGGAGTGGCGAGAGATGAAATTGGGCT

TGCCGGCGGCAGAGCGATCCACTGAGGGTGAGCACCGTCTCCGACGCCTCAAGCCACTAC

CAGACCCCCGCATCCATGCCTATACTCATTGCGAGATTCATCCGGCTATGATTCTCGGTA

TCTGTGCAAGTATCATTCCCTTCCCCGATCACAACCATCGCCCCTAACTTTT

>4C-F *Alternaria alternata/arborescens*, *rpb2* gene

TGGATTGCNGAACCNTTGGTCGCCACTTGNTTCCGTATCCTCTTCCTGAAGCTCACCAAG

GATGTGTACAAGTACCTCCAGCGATGTGTTGAGAACAACCAGGATTTCAACGTTCAGATG

GCTGTCAAGGCCAGCATCATCACAAACGGCCTGAAATACTCCCTGGCAACAGGAAACTGG

GGTGACCAGAAGAAGGCCGCTTCCGCGAAAGCCGGTGTCTCCCAGGTGTTGAACCGATAC

ACTTACGCCTCCACACTGTCACATTTGCGTCGAACGAATACCCCTGTTGGTCGTGATGGA

AAGCTGGCCAAGCCGCGACAACTCCACAACTCTCATTGGGGTCTTGTCTGCCCTGCCGAG

ACTCCTGAAGGACAGGCTTGTGGTCTGGTTAAGAACCTGTCTTTGATGTGCTATGTTAGT

GTCGGTAGTGACGCTTCTCCCATCATCGACTTCATGACACAACGAAACATGCAACTCCTA

GAAGAGTATGATCAGAACCAGAACCCCGATGCCACCAAGGTCTTTGTCAACGGTGTCTGG

GTTGGTGTCCATTCCAACGCTCAACAACTTGTCACAGTTGTGCAGGAGCTGCGACGAAAC

GGAACTCTATCCTATGAGATGAGTCTGATTCGTGACATTCGTGACCGAGAGTTCAAGATC

TTCACAGATGCTGGCCGTGTCATGAGACCACTGTTCGTTGTTGAGAACGACATTCGGAAG

CCAAACCGCAACCACCTCATCTTCACCAAGGAGATCAGTAACAAGCTCAAGCAGGAACAA

CAAGAGACAAGCACACGACAGGGTTGGAGCCAGGATGAGGTCGAATCAGCTACCTACGGC

TGGAGAGGTCTTATTCAAGACGGTGTTGTGGAGTACCTAGACGCTGAAGAGGAGGAGACC

GCCATGATAACGTTCTCCCCTGAGGATCTGGAGGAGTGGCGAGAGATGAAATTGGGTTTG

CCGGCGGCGGAGCGATCCACCGAGGGTGAGCACCGTCTCCGAGGCCTCAAGCCACTACCA

GACCCCCGCATCCATGCCTATACTCATTGCGAGATTCATCCGGCTATGATTCTCGGTATC

TGTGCCAGTATCATTCCCTCCCCGATCACAACCATCGCCCCTAACTTCT

>5 *Alternaria alternata/arborescens*, *rpb2* gene

TGGATTGCNGGACCCTTGGGTCGCCACTTGTTCCGTATCCTCTTCCTGAAGCTCACCAAG

GATGTGTACAAGTACCTCCAGCGATGTGTTGAGAACAACCAGGATTTCAACGTTCAGATG

GCTGTCAAGGCCAGCATCATCACAAACGGCCTGAAATACTCCCTGGCAACAGGAAACTGG

GGTGACCAGAAGAAGGCCGCTTCCGCGAAAGCCGGTGTYTCCCAGGTGTTGAACCGATAC

ACTTACGCCTCCACACTGTTCACATTTGCGTCGAAACGAATACCCCTGTTGGTCGTGATG

GAAAGCTGGCCAAGCCGCGACAACTCCACAACTCTCATTGGGGTCTTGTCTGCCCTGCCG

AGACTCCTGAAGGACAGGCTTGTGGTCTGGTTAAGAACCTGTCTTTGATGTGCTATGTTA

GTGTCGGTAGTGACGCTTCTCCCATCATCGACTTCATGACACAACGAAACATGCAACTCC

TAGAAGAGTATGATCAGAACCAGAACCCCGATGCCACCAAGGTCTTTGTCAACGGTGTCT

GGGTTGGTGTCCATTCCAACGCTCAACAACTTGTCACAGTTGTGCAGGAGCTGCGACGAA

ACGGAACTCTATCCTATGAGATGAGTCTGATTCGTGACATTCGTGACCGAGAGTTCAAGA

TCTTCACAGATGCTGGCCGTGTCATGAGACCACTGTTCGTTGTTGAGAACGACATTCGGA

AGCCAAACCGCAACCACCTCATCTTCACCAAGGAGATCAGTAACAAGCTCAAGCAGGAAC

AACAAGAGACAAGCACACGACAGGGTTGGAGCCAGGATGAGGTCGAATCAGCTACCTACG

GCTGGAGAGGTCTTATTCAAGACGGTGTTGTGGAGTACCTAGACGCTGAAGAGGAGGAGA

CCGCCATGATAACGTTCTCCCCTGAGGATCTGGAGGAGTGGCGAGAGATGAAATTGGGTT

TGCCGGCGGCGGAGCGATCCACCGAGGGTGAGCACCGTCTCCGACGCCTCAAGCCACTAC

CAGACCCCCGCATCCATGCCTATACTCATTGCGAGATTCATCCGGCTATGATTCTCGGTA

TCTGTGCCAGTATCATTCCCTCCCCGATCACAACCATCGCCCCCTAACCTTT

>5C *Alternaria ovoidea*, *rpb2* gene

TGCGGACCNTTGGTCGCCANCTTGTTCCGTATCCTCTTCCTGAAGCTCACCAAGGATGTG

TACAAGTTACCTCCAGCGATGTGTTGAGAACAACCAGGATTTCAACGTTCAGATGGCTGT

CAAGGCCAGCATCATCACAAACGGCCTGAAATACTCCCTGGCAACAGGAAACTGGGGTGA

CCAGAAGAAGGCCGCTTCCGCGAAAGCCGGTGTCTCCCAGGTGTTGAACCGATACACTTA

CGCCTCCACACTGTCACATTTGCGTCGAACGAATACCCCTGTTGGTCGTGATGGAAAGCT

GGCCAAGCCGCGACAACTCCACAACTCTCATTGGGGTCTTGTCTGCCCTGCCGAGACTCC

TGAAGGACAGGCTTGTGGTCTGGTTAAGAACCTGTCTTTGATGTGCTATGTTAGTGTCGG

TAGTGACGCTTCTCCCATCATCGACTTCATGACACAACGAAACATGCAACTCCTAGAAGA

GTATGATCAGAACCAGAACCCCGATGCCACCAAGGTCTTTGTCAACGGTGTCTGGGTTGG

TGTCCATTCCAACGCTCAACAACTTGTCACAGTTGTGCAGGAGCTGCGACGAAACGGAAC

TCTATCCTATGAGATGAGTCTGATTCGTGACATTCGTGACCGAGAGTTCAAGATCTTCAC

AGATGCTGGCCGTGTCATGAGACCACTGTTCGTTGTTGAGAACGACATTCGGAAGCCAAA

CCGCAACCACCTCATCTTCACCAAGGAGATCAGTAACAAGCTCAAGCAGGAACAACAAGA

GACAAGCACACGACAGGGTTGGAGCCAGGATGAGGTCGAATCAGCTACCTACGGCTGGAG

AGGTCTTATTCAAGACGGTGTTGTGGAGTACCTAGACGCTGAAGAGGAGGAGACCGCCAT

GATAACGTTCTCCCCTGAGGATCTGGAGGAGTGGCGAGAGATGAAATTGGGTTTGCCGGC

GGCGGAGCGATCCACCGAGGGTGAGCACCGTCTCCGACGCCTCAAGCCACTACCAGACCC

CCGCATCCATGCCTATACTCATTGCGAGATTCATCCGGCTATGATTCTCGGTATCTGTGC

CAGTATCATTCCCTTCCCCGATCACAACCATCGCCCCTAACTTTT

>6 *Alternaria alternata/arborescens*, *rpb2* gene

TGGATTGCNGGACCCTTGGTCGCCACTTGTTCCGTATCCTCTTCCTGAAGCTCACCAAGG

ATGTGTACAAGTTACCTCCAGCGATGTGTTGAGAACAACCAGGATTTCAACGTTCAGATG

GCTGTCAAGGCCAGCATCATCACAAACGGCCTGAAATACTCCCTGGCAACAGGAAACTGG

GGTGACCAGAAGAAGGCCGCTTCCGCGAAAGCCGGTGTCTCCCAGGTGTTGAACCGATAC

ACTTACGCCTCCACACTGTCACATTTGCGTCGAACGAATACCCCTGTTGGTCGTGATGGA

AAGCTGGCCAAGCCGCGACAACTCCACAACTCTCATTGGGGTCTTGTCTGCCCTGCCGAG

ACTCCTGAAGGACAGGCTTGTGGTCTGGTTAAGAACCTGTCTTTGATGTGCTATGTTAGT

GTCGGTAGTGACGCTTCTCCCATCATCGACTTCATGACACAACGAAACATGCAACTCCTA

GAAGAGTATGATCAGAACCAGAACCCCGATGCCACCAAGGTCTTTGTCAACGGTGTCTGG

GTTGGTGTCCATTCCAACGCTCAACAACTTGTCACAGTTGTGCAGGAGCTGCGACGAAAC

GGAACTCTATCCTATGAGATGAGTCTGATTCGTGACATTCGTGACCGAGAGTTCAAGATC

TTCACAGATGCTGGCCGTGTCATGAGACCACTGTTCGTTGTTGAGAACGACATTCGGAAG

CCAAACCGCAACCACCTCATCTTCACCAAGGAGATCAGTAACAAGCTCAAGCAGGAACAA

CAAGAGACAAGCACACGACAGGGTTGGAGCCAGGATGAGGTCGAATCAGCTACCTACGGC

TGGAGAGGTCTTATTCAAGACGGTGTTGTGGAGTACCTAGACGCTGAAGAGGAGGAGACC

GCCATGATAACGTTCTCCCCTGAGGATCTGGAGGAGTGGCGAGAGATGAAATTGGGTTTG

CCGGCGGCGGAGCGATCCACCGAGGGTGAGCACCGTCTCCGACGCCTCAAGCCACTACCA

GACCCCCGCATCCATGCCTATACTCATTGCGAGATTCATNCGGNTATGATTCTCGGTATC

TGTGCCAGTATCATTCCCTCCCCGATCACAACCATCGCCCCNACCTCT

>6C *Alternaria ovoidea*, *rpb2* gene

AATGNNTTGCTGGACCTTGGTCGCCACTTGTTCCGTATCCTCTTCCTGAAGCTCACCAAG

GAACGTATACAAGTACCTTCCAGCGATGTGTTGAGAAACAACCAGGATTTCAACGTTCAG

ATGGCTGTCAAGGCCAGCWTCATCACAAACGGCCTGAAATACTCCCTGGCAACAGGAAAC

TGGGGTGACCAGAAGAAGGCCGCTTCCGCGAAAGCCGGTGTCTCCCAGGTGTTGAACCGA

TACACTTACGCCTCCACACTGTCCCATTTGCGTCGAACGAATACCCCTGTTGGTCGTGAT

GGAAAGCTGGCCAAGCCGCGACAACTCCACAACTCTCATTGGGGTCTTGTCTGCCCTGCC

GAGACTCCTGAAGGACAGGCTTGTGGTCTGGTTAAGAACCTGTCTTTGATGTGCTATGTT

AGTGTCGGTAGTGACGCTTCTCCCATCATCGACTTCATGACACAACGAAACATGCAACTT

CTAGAAGAGTATGATCAGAACCAGAACCCCGATGCCACCAAGGTCTTCGTCAACGGTGTC

TGGGTTGGTGTCCATTCCAACGCTCAACAACTTGTCACAGTTGTGCAGGAGCTGCGACGA

AACGGAACTCTATCCTATGAGATGAGTCTGATTCGTGACATTCGTGACCGAGAGTTCAAG

ATCTTCACAGATGCTGGCCGTGTCATGAGACCACTGTTCGTTGTTGAGAACGACATTCGG

AAGCCAAACCGCAACCACCTCATCTTCACCAAGGAGATCAGTAACAAGCTCAAGCAGGAG

CAACAAGAGACAAGCACACGACAGGGTTGGAGCCAGGATGAGGTCGAATCAGCTACCTAC

GGCTGGAGAGGTCTCATTCAAGACGGTGTTGTGGAGTACCTTGACGCTGAAGAGGAGGAG

ACCGCCATGATAACGTTCTCCCCCTGAGGATCTGGGARGAGTGGCGARAGATGAAATTGG

GGCTTGCCGGCGGCGGAGCGATYCACCGNAGGGTGAAGCACCGTCTTCCGACGCCTCAAG

CCACTACCAGACCCCCGCATCCATGCCTATACTCATTGCGAGATTCATCCGGCTATGATT

CTCGGTATCTGTGCCAGTATCATTCCCTCCCCGATCACAACCATCGCCCCCTAACCTTT

>7 *Alternaria alternata/arborescens*, *rpb2* gene

TTGCGGNACCNTTGGGTCGCCACCTTGNTTCCGGTATCCTCTTCCTGGAAGCTCACCAAG

GAACGTANCCNNTTTACCTCCAGCGAATGTTGTTTGAGAACAACCAGGATTTCAACGTTC

AGATGGCTGTCAAGGCCAGTATCATCACAAATGGCCTGAAATACTCCCTGGCAACAGGAA

ACTGGGGTGACCAGAAGAAGGCCGCGTCCGCGAAAGCCGGTGTCTCTCAGGTGTTGAACC

GATACACTTACGCCTCCACACTGTCCCATTTGCGTCGAACGAATACCCCTGTTGGTCGTG

ATGGAAAGCTGGCCAAGCCGCGACAACTCCACAACTCTCATTGGGGTCTTGTCTGCCCTG

CCGAGACTCCTGAAGGACAGGCTTGTGGTTTGGTTAAGAACCTGTCTTTGATGTGCTATG

TTAGTGTCGGTAGTGACGCTTCTCCCATCATCGACTTCATGACACAACGAAACATGCAAC

TTCTAGAAGAGTATGATCAGAACCAGAACCCCGATGCCACCAAGGTCTTCGTCAATGGTG

TCTGGGTTGGTGTCCATTCCAACGCTCAACAACTTGTCACAGTTGTGCAGGAGCTGCGAC

GAAACGGAACTCTATCCTATGAGATGAGTCTGATTCGTGACATTCGTGACCGAGAGTTCA

AGATCTTCACAGATGCTGGCCGTGTCATGAGACCACTGTTCGTTGTTGAGAACGACATTC

GGAAGCCAAACCGCAACCACCTCATCTTCACCAAGGAGATCAGTAACAAGCTCAAGCAGG

AGCAACAAGAGACAAGCACACGACAGGGTTGGAGCCAGGATGAGGTCGAATCAGCTACCT

ACGGCTGGAGAGGTCTTATTCAAGACGGTGTTGTGGAGTACCTAGACGCTGAAGAGGAGG

AGACCGCCATGATAACGTTCTCCCCTGAGGATCTGGAGGAGTGGCGAGAGATGAAGTTGG

GCTTGCCGGCGGCGGAGCGATCCACCGAAGGTGAGCACCGTCTCCGACGCCTCAAGCCAC

TACCAGACCCCCGCATCCATGCCTATACACATTGCGAGATTCATCCGGCTATGATTCTCG

GTATCTGTGCCAGTATCATTCCCTTCCCCGATCACAACCATCGCCCCNANTTTTCCT

>7C *Alternaria* species, *rpb2* gene

TGGATTGCGGACCCTTGGTCGCCACTTGTTCCGTATCCTCTTCCTGAAGCTCACCAAGGA

TGTGTACAAGTACCTCCAGCGATGTGTTTGAGAACAACCAGGATTTCAACGTTCAGATGG

CTGTCAAGGCCAGCATCATCACAAACGGCCTGAAATACTCCCTGGCAACAGGAAACTGGG

GTGACCAGAAGAAGGCCGCTTCCGCGAAAGCCGGTGTCTCCCAGGTGTTGAACCGATACA

CTTACGCCTCCACACTGTCACATTTGCGTCGAACGAATACCCCTGTTGGTCGTGATGGAA

AGCTGGCCAAGCCGCGACAACTCCACAACTCTCATTGGGGTCTTGTCTGCCCTGCCGAGA

CTCCTGAAGGACAGGCTTGTGGTCTGGTTAAGAACCTGTCTTTGATGTGCTATGTTAGTG

TCGGTAGTGACGCTTCTCCCATCATCGACTTCATGACACAACGAAACATGCAACTCCTAG

AAGAGTATGATCAGAACCAGAACCCCGATGCCACCAAGGTCTTTGTCAACGGTGTCTGGG

TTGGTGTCCATTCCAACGCTCAACAACTTGTCACAGTTGTGCAGGAGCTGCGACGAAACG

GAACTCTATCCTATGAGATGAGTCTGATTCGTGACATTCGTGACCGAGAGTTCAAGATCT

TCACAGATGCTGGCCGTGTCATGAGACCACTGTTCGTTGTTGAGAACGACATTCGGAAGC

CAAACCGCAACCACCTCATCTTCACCAAGGAGATCAGTAACAAGCTCAAGCAGGAACAAC

AAGAGACAAGCACACGACAGGGTTGGAGCCAGGATGAGGTCGAATCAGCTACCTACGGCT

GGAGAGGTCTTATTCAAGACGGTGTTGTGGAGTACCTAGACGCTGAAGAGGAGGAGACCG

CCATGATAACGTTCTCCCCTGAGGATCTGGAGGAGTGGCGAGAGATGAAATTGGGTTTGC

CGGCGGCGGAGCGATCCACCGAGGGTGAGCACCGTCTCCGACGCCTCAAGCCACTACCAG

ACCCCCGCATCCATGCCTATACTCATTGCGAGATTCATAANGGGTATGATTCTCGGTATC

TGTGCCAGTATCATTCCCTCCCCGATCACAACCATCGCCCTACCTCT

>8 *Alternaria alternata/arborescens*, *rpb2* gene

TGGATTGCTGGACCCTTGGTCGCCACTTGTTCCGTATCCTCTTCCTGAAGCTCACCAAGG

AACGTATACAAGTTACCTCCAGCGATGTGTTGAGAACAACCAGGATTTCAACGTTCAGAT

GGCTGTCAAGGCCAGCATCATCACAAACGGCCTGAAATACTCCCTGGCAACAGGAAACTG

GGGTGACCAGAAGAAGGCCGCTTCCGCGAAAGCCGGTGTCTCCCAGGTGTTGAACCGATA

CACTTACGCCTCCACACTGTCCCATTTGCGTCGAACGAATACCCCTGTTGGTCGTGATGG

AAAGCTGGCCAAGCCGCGACAACTCCACAACTCTCATTGGGGTCTTGTCTGCCCTGCCGA

GACTCCTGAAGGACAGGCTTGTGGTCTGGTTAAGAACCTGTCTTTGATGTGCTATGTTAG

TGTCGGTAGTGACGCTTCTCCCATCATCGACTTCATGACACAACGAAACATGCAACTTCT

AGAAGAGTATGATCAGAACCAGAACCCCGATGCCACCAAGGTCTTCGTCAACGGTGTCTG

GGTTGGTGTCCATTCCAACGCTCAACAACTTGTCACAGTTGTGCAGGAGCTGCGACGAAA

CGGAACTCTATCCTATGAGATGAGTCTGATTCGTGACATTCGTGACCGAGAGTTCAAGAT

CTTCACAGATGCTGGCCGTGTCATGAGACCACTGTTCGTTGTTGAGAACGACATTCGGAA

GCCAAACCGCAACCACCTCATCTTCACCAAGGAGATCAGTAACAAGCTCAAGCAGGAGCA

ACAAGAGACAAGCACACGACAGGGTTGGAGCCAGGATGAGGTCGAATCAGCTACCTACGG

CTGGAGAGGTCTCATTCAAGACGGTGTTGTGGAGTACCTTGACGCTGAAGAGGAGGAGAC

CGCCATGATAACGTTCTCCCCTGAGGATCTGGAGGAGTGGCGAGAGATGAAATTGGGCTT

GCCGGCGGCGGAGCGATCCACCGAGGGTGAGCACCGTCTCCGACGCCTCAAGCCACTACC

AGACCCCCGCATCCATGCCTATACTCATTGCGAGATTCATCCGGCTATGATTCTCGGTAT

CTGTGCCAGTATCATTCCCTTCCCCGATCACAACCATCGCCCCTAACCTTT

>8-C *Alternaria alternata/arborescens*, *rpb2* gene

TGGGANTGCGGACCTTGGTCGCCAACTTGTTCCGTATCCTCTTCCTGAAGCTCACCAAGG

ATGTGTACAAGTTACCTCCAGCGATGTGTTTGAGAACAACCAGGATTTCAACGTTCAGAT

GGCTGTCAAGGCCAGCATCATCACAAACGGCCTGAAATACTCCCTGGCAACAGGAAACTG

GGGTGACCAGAAGAAGGCCGCTTCCGCGAAAGCCGGTGTCTCCCAGGTGTTGAACCGATA

CACTTACGCCTCCACACTGTCACATTTGCGTCGAACGAATACCCCTGTTGGTCGTGATGG

AAAGCTGGCCAAGCCGCGACAACTCCACAACTCTCATTGGGGTCTTGTCTGCCCTGCCGA

GACTCCTGAAGGACAGGCTTGTGGTCTGGTTAAGAACCTGTCTTTGATGTGCTATGTTAG

TGTCGGTAGTGACGCTTCTCCCATCATCGACTTCATGACACAACGAAACATGCAACTCCT

AGAAGAGTATGATCAGAACCAGAACCCCGATGCCACCAAGGTCTTTGTCAACGGTGTCTG

GGTTGGTGTCCATTCCAACGCTCAACAACTTGTCACAGTTGTGCAGGAGCTGCGACGAAA

CGGAACTCTATCCTATGAGATGAGTCTGATTCGTGACATTCGTGACCGAGAGTTCAAGAT

CTTCACAGATGCTGGCCGTGTCATGAGACCACTGTTCGTTGTTGAGAACGACATTCGGAA

GCCAAACCGCAACCACCTCATATTCACCAAGGAGATCAGTAACAAGCTCAAGCAGGAACA

ACAAGAGACAAGCACACGACAGGGTTGGAGCCAGGATGAGGTCGAATCAGCTACCTACGG

CTGGAGAGGTCTTATTCAAGACGGTGTTGTGGAGTACCTAGACGCTGAAGAGGAGGAGAC

CGCCATGATACGTTCTCCCCTGAGGATCTGGARGAGTGGCGARAGAWKAAATTGGGKTTG

CCGGCGGCGGGASCGATCCACCGAGGGTGAGCACCGTCTCCGACGCCTCAAGCCACTACC

AGACCCCCGCATCCATGCCTATACTCATTGCGAGATTCTTAAAGGGGTATGATTCTCGGT

ATCTGTGCCAGTATCATTCCCTCCCCGATCACAACCATCGCCCCNACCTTT

>9-F *Alternaria alternata/arborescens*, *rpb2* gene

ATGGANTGCGGACCTGGTCGCCACTNGTTCCGTATCCTCTTCCTGAAGCTCACCAAGGAT

GTGTCCANTTTACCTCCAGCGATGTGTTGAGAACAACCAGGATTTCAACGTTCAGATGGC

TGTCAAGGCCAGCATCATCACAAACGGCCTGAAATACTCCCTGGCAACAGGAAACTGGGG

TGACCAGAAGAAGGCCGCTTCCGCGAAAGCCGGTGTCTCCCAGGTGTTGAACCGATACAC

TTACGCCTCCACACTGTCACATTTGCGTCGAACGAATACCCCTGTTGGTCGTGATGGAAA

GCTGGCCAAGCCGCGACAACTCCACAACTCTCATTGGGGTCTTGTCTGCCCTGCCGAGAC

TCCTGAAGGACAGGCTTGTGGTCTGGTTAAGAACCTGTCTTTGATGTGCTATGTTAGTGT

CGGTAGTGACGCTTCTCCCATCATCGACTTCATGACACAACGAAACATGCAACTCCTAGA

AGAGTATGATCAGAACCAGAACCCCGATGCCACCAAGGTCTTTGTCAACGGTGTCTGGGT

TGGTGTCCATTCCAACGCTCAACAACTTGTCACAGTTGTGCAGGAGCTGCGACGAAACGG

AACTCTATCCTATGAGATGAGTCTGATTCGTGACATTCGTGACCGAGAGTTCAAGATCTT

CACAGATGCTGGCCGTGTCATGAGACCACTGTTCGTTGTTGAGAACGACATTCGGAAGCC

AAACCGCAACCACCTCATCTTCACCAAGGAGATCAGTAACAAGCTCAAGCAGGAACAACA

AGAGACAAGCACACGACAGGGTTGGAGCCAGGATGAGGTCGAATCAGCTACCTACGGCTG

GAGAGGTCTTATTCAAGACGGTGTTGTGGAGTACCTAGACGCTGAAGAGGAGGAGACCGC

CATGATAACGTTCTCCCCTGAGGATCTGGAGGAGTGGCGAGAGATGAAATTGGGTTTGCC

GGCGGCGGAGCGATCCACCGAGGGTGAGCACCGTCTCCGACGCCTCAAGCCACTACCAGA

CCCCCGCATCCATGCCTATACTCATTGCGAGATTCATCCGGCTATGATTCTCGGTATCTG

TGCCAGTATCATTCCCTTCCCCGATCACAACCATCGCCCCTAACCTCT

>FC4 *Alternaria ovoidea*, *rpb2* gene

CCTTGGTTCGCCACTTGTTCCGTATCCTCTTCCTGAAGCTCACCAAGGACGTATACAAGT

TACCTCCAGCGATGTGTTGAGAACAACCAGGATTTCAACGTTCAGATGGCTGTCAAGGCC

AGCATCATCACAAACGGCCTGAAATACTCCCTGGCAACAGGAAACTGGGGTGACCAGAAG

AAGGCCGCTTCCGCGAAAGCCGGTGTCTCCCAGGTGTTGAACCGATACACTTACGCCTCC

ACACTGTCCCATTTGCGTCGAACGAATACCCCTGTTGGTCGTGATGGAAAGCTGGCCAAG

CCGCGACAACTCCACAACTCTCATTGGGGTCTTGTCTGCCCTGCCGAGACTCCTGAAGGA

CAGGCTTGTGGTCTGGTTAAGAACCTGTCTTTGATGTGCTATGTTAGTGTCGGTAGTGAC

GCTTCTCCCATCATCGACTTCATGACACAACGAAACATGCAACTTCTAGAAGAGTATGAT

CAGAACCAGAACCCCGATGCCACCAAGGTCTTCGTCAACGGTGTCTGGGTTGGTGTCCAT

TCCAACGCTCAACAACTTGTCACAGTTGTGCAGGAGCTGCGACGAAACGGAACTCTATCC

TATGAGATGAGTCTGATTCGTGACATTCGTGACCGAGAGTTCAAGATCTTCACAGATGCT

GGCCGTGTCATGAGACCACTGTTCGTTGTTGAGAACGACATTCGGAAGCCAAACCGCAAC

CACCTCATCTTCACCAAGGAGATCAGTAACAAGCTCAAGCAGGAGCAACAAGAGACAAGC

ACACGACAGGGTTGGAGCCAGGATGAGGTCGAATCAGCTACCTACGGCTGGAGAGGTCTC

ATTCAAGACGGTGTTGTGGAGTACCTTGACGCTGAAGAGGAGGAGACCGCCATGATAACG

TTCTCCCCTGAGGATCTGGAGGAGTGGCGAGAGATGAAATTGGGCTTGCCGGCGGCGGAG

CGATCCACCGAGGGTGAGCACCGTCTCCGACGCCTCAAGCCACTACCAGACCCCCGCATC

CATGCCTATACTCATTGCGAGATTCATCCGGCTATGATTCTCGGTATCTGTGCCAGTATC

ATTCCCTCCCCGATCACAACCATCGCCCCTAACCTCTC

>F12 *Alternaria alternata/arborescens*, *rpb2* gene

TGGANTGCTGGACCTTTGGTCGCCACTTGTTCCGTATCCTCTTCCTGAAGCTCACCAAGG

ACGTATACAAGTACCTCCAGCGATGTGTTGAGAACAACCAGGATTTCAACGTTCAGATGG

CTGTCAAGGCCAGCATCATCACAAACGGCCTGAAATACTCCCTGGCAACAGGAAACTGGG

GTGACCAGAAGAAGGCCGCTTCCGCGAAAGCCGGTGTCTCCCAGGTGTTGAACCGATACA

CTTACGCCTCCACACTGTCCCATTTGCGTCGAACGAATACCCCTGTTGGTCGTGATGGAA

AGCTGGCCAAGCCGCGACAACTCCACAACTCTCATTGGGGTCTTGTCTGCCCTGCCGAGA

CTCCTGAAGGACAGGCTTGTGGTCTGGTTAAGAACCTGTCTTTGATGTGCTATGTTAGTG

TCGGTAGTGACGCTTCTCCCATCATCGACTTCATGACACAACGAAACATGCAACTTCTAG

AAGAGTATGATCAGAACCAGAACCCCGATGCCACCAAGGTCTTCGTCAACGGTGTCTGGG

TTGGTGTCCATTCCAACGCTCAACAACTTGTCACAGTTGTGCAGGAGCTGCGACGAAACG

GAACTCTATCCTATGAGATGAGTCTGATTCGTGACATTCGTGACCGAGAGTTCAAGATCT

TCACAGATGCTGGCCGTGTCATGAGACCACTGTTCGTTGTTGAGAACGACATTCGGAAGC

CAAACCGCAACCACCTCATCTTCACCAAGGAGATCAGTAACAAGCTCAAGCAGGAGCAAC

AAGAGACAAGCACACGACAGGGTTGGAGCCAGGATGAGGTCGAATCAGCTACCTACGGCT

GGAGAGGTCTCATTCAAGACGGTGTTGTGGAGTACCTTGACGCTGAAGAGGAGGAGACCG

CCATGATAACGTTCTCCCCTGAGGATCTGGAGGAGTGGCGAGAGATGAAATTGGGCTTGC

CGGCGGCGGAGCGATCCACCGAGGGTGAGCACCGTCTCCGACGCCTCAAGCCACTACCAG

ACCCCCGCATCCATGCCTATACTCATTGCGAGATTCATCCGGCTATGATTCTCGGTATCT

GTGCCAGTATCATTCCCTTCCCCGATCACAACCATCGCCCCTAACNTTT

>F2C-F *Alternaria alternata/arborescens*, *rpb2* gene

ATGGNTTGCGGANCTTGGTCGCCACTTGTTCCGTATCCTCTTCCTGAAGCTCACCAAGGA

TGTGTCCNNTTACCTCCAGCGATGTGTTGAGAACAACCAGGATTTCAACGTTTCAGATGG

CTGTCAAGGCCAGCATCATCACAAACGGCCTGAAATACTCCCTGGCAACAGGAAACTGGG

GTGACCAGAAGAAGGCCGCTTCCGCGAAAGCCGGTGTCTCCCAGGTGTTGAACCGATACA

CTTACGCCTCCACACTGTCCCATTTGCGTCGAACGAATACCCCTGTTGGTCGTGATGGAA

AGCTGGCCAAGCCGCGACAACTCCACAACTCTCATTGGGGTCTTGTCTGCCCTGCCGAGA

CTCCTGAAGGACAGGCTTGTGGTCTGGTTAAGAACCTGTCTTTGATGTGCTATGTTAGTG

TCGGTAGTGACGCTTCTCCCATCATCGACTTCATGACACAACGAAACATGCAACTTCTAG

AAGAGTATGATCAGAACCAGAACCCCGATGCCACCAAGGTCTTCGTCAACGGTGTCTGGG

TTGGTGTCCATTCCAACGCTCAACAACTTGTCACAGTTGTGCAGGAGCTGCGACGAAACG

GAACTCTATCCTATGAGATGAGTCTGATTCGTGACATTCGTGACCGAGAGTTCAAGATCT

TCACAGATGCTGGCCGTGTCATGAGACCACTGTTCGTTGTTGAGAACGACATTCGGAAGC

CAAACCGCAACCACCTCATCTTCACCAAGGAGATCAGTAACAAGCTCAAGCAGGAGCAAC

AAGAGACAAGCACACGACAGGGTTGGAGCCAGGATGAGGTCGAATCAGCTACCTACGGCT

GGAGAGGTCTCATTCAAGACGGTGTTGTGGAGTACCTTGACGCTGAAGAGGAGGAGACCG

CCATGATAACGTTCTCCCCTGAGGATCTGGAGGAGTGGCGAGAGATGAAATTGGGCTTGC

CGGCGGCGGAGCGATCCACCGAGGGTGAGCACCGTCTCCGACGCCTCAAGCCACTACCAG

ACCCCCGCATCCATGCCTATACTCATTGCGAGATTCATCCGGCTATGATTCTCGGTATCT

GTGCCAGTATCATTCCCTTCCCCGATCACAACCATCGCCCCTAACCTTT

>F3-F *Alternaria alternata/arborescens*, *rpb2* gene

TGGANTTGCGGACCCTTGGTCGCCACTTGTTCCGTATCCTCTTCCTGAAGCTCACCAAGG

ACGTATACAAGTACCTCCAGCGATGTGTTGAGAACAACCAGGATTTCAACGTTCAGATGG

CTGTCAAGGCCAGCATCATCACAAATGGCCTGAAATACTCCCTGGCAACAGGAAACTGGG

GTGACCAGAAGAAGGCCGCGTCCGCGAAAGCCGGTGTCTCTCAGGTGTTGAACCGATACA

CTTACGCCTCCACACTGTCCCATTTGCGTCGAACGAATACCCCTGTTGGTCGTGATGGAA

AGCTGGCCAAGCCGCGACAACTCCACAACTCTCATTGGGGTCTTGTCTGCCCTGCCGAGA

CTCCTGAAGGACAGGCTTGTGGTTTGGTTAAGAACCTGTCTTTGATGTGCTATGTTAGTG

TCGGTAGTGACGCTTCTCCCATCATCGACTTCATGACACAACGAAACATGCAACTTCTAG

AAGAGTATGATCAGAACCAGAACCCCGATGCCACCAAGGTCTTCGTCAATGGTGTCTGGG

TTGGTGTCCATTCCAACGCTCAACAACTTGTCACAGTTGTGCAGGAGCTGCGACGAAACG

GAACTCTATCCTATGAGATGAGTCTGATTCGTGACATTCGTGACCGAGAGTTCAAGATCT

TCACAGATGCTGGCCGTGTCATGAGACCACTGTTCGTTGTTGAGAACGACATTCGGAAGC

CAAACCGCAACCACCTCATCTTCACCAAGGAGATCAGTAACAAGCTCAAGCAGGAGCAAC

AAGAGACAAGCACACGACAGGGTTGGAGCCAGGATGAGGTCGAATCAGCTACCTACGGCT

GGAGAGGTCTTATTCAAGACGGTGTTGTGGAGTACCTAGACGCTGAAGAGGAGGAGACCG

CCATGATAACGTTCTCCCCTGAGGATCTGGAGGAGTGGCGAGAGATGAAGTTGGGCTTGC

CGGCGGCGGAGCGATCCACCGAAGGTGAGCACCGTCTCCGACGCCTCAAGCCACTACCAG

ACCCCCGCATCCATGCCTATACACATTGCGAGATTCATCCGGCTATGATTCTCGGTATTC

TGTGCCAGTATCATTCCCTTCCCCGATCACAACCATCGCCCCTACTTTTCC

>F5C-F *Alternaria ovoidea*, *rpb2* gene

TGGANTTGCNGGACCCTTGGTCGCCACTTGNTTCCGTATCCTCTTCCTGAAGCTCACCAA

GGATGTGTACAAGTTACCTCCAGCGATGTGTTGAGAACAACCAGGATTTCAACGTTCAGA

TGGCTGTCAAGGCCAGCATCATCACAAACGGCCTGAAATACTCCCTGGCAACAGGAAACT

GGGGTGACCAGAAGAAGGCCGCTTCCGCGAAAGCCGGTGTCTCCCAGGTGTTGAACCGAT

ACACTTACGCCTCCACACTGTCACATTTGCGTCGAACGAATACCCCTGTTGGTCGTGATG

GAAAGCTGGCCAAGCCGCGACAACTCCACAACTCTCATTGGGGTCTTGTCTGCCCTGCCG

AGACTCCTGAAGGACAGGCTTGTGGTCTGGTTAAGAACCTGTCTTTGATGTGCTATGTTA

GTGTCGGTAGTGACGCTTCTCCCATCATCGACTTCATGACACAACGAAACATGCAACTCC

TAGAAGAGTATGATCAGAACCAGAACCCCGATGCCACCAAGGTCTTTGTCAACGGTGTCT

GGGTTGGTGTCCATTCCAACGCTCAACAACTTGTCACAGTTGTGCAGGAGCTGCGACGAA

ACGGAACTCTATCCTATGAGATGAGTCTGATTCGTGACATTCGTGACCGAGAGTTCAAGA

TCTTCACAGATGCTGGCCGTGTCATGAGACCACTGTTCGTTGTTGAGAACGACATTCGGA

AGCCAAACCGCAACCACCTCATCTTCACCAAGGAGATCAGTAACAAGCTCAAGCAGGAAC

AACAAGAGACAAGCACACGACAGGGTTGGAGCCAGGATGAGGTCGAATCAGCTACCTACG

GCTGGAGAGGTCTTATTCAAGACGGTGTTGTGGAGTACCTAGACGCTGAAGAGGAGGAGA

CCGCCATGATAACGTTCTCCCCTGAGGATCTGGAGGAGTGGCGAGAGATGAAATTGGGTT

TGCCGGCGGCGGAGCGATCCACCGAGGGTGAGCACCGTCTCCGACGCCTCAAGCCACTAC

CAGACCCCCGCATCCATGCCTATACTCATTGCGAGATTCATCCGGCTATGATTCTCGGTA

TCTGTGCCAGTATCATTCCCTCCCCGATCACAACCATCGCCCCTAACTTTT
