# Supplementary material for: Potential Biocontrol Agents of Corn Tar Spot Disease Isolated from Overwintered Phyllachora maydis Stromata
Source: Microorganisms. 2023 Jun 10;11(6):1550. doi: 10.3390/microorganisms11061550 (PMC10303863; doi:10.3390/microorganisms11061550)

Supplementary File S3

A recent study that identified several new *Alternaria* species suggested that portions of three genes (*Alt-a1, gapdh,* and *rpb2*) were sufficiently divergent to identify *Alternaria* to the species level (Li et al. 2022, reference 18 in the manuscript). Therefore, we amplified portions of these three genes from genomic DNA from our 22 *Alternaria* fungi. Phylogenetic analysis of the multilocus alignment of the three gene sequences amplified from our *Alternaria* collection and sequences of 28 *Alternaria* ex-type species (members of the *Alternaria* section, obtained from the NCBI) is shown in the figure on the next page. Four *Alternaria* ex-type species used in the Li et al. (2022) study were not included in the current analysis because there were no sequences in NCBI for one or more of the three genes (*Alt-a1, gapdh,* and *rpb2*). In addition, the partial *rpb2* gene sequence from *A. betae-kenyensis* was much shorter than other *rpb2* sequences in the alignment and therefore *A. betae-kenyensis* was removed from the analysis. There was good bootstrap support (99) to suggest that six of our *Alternaria* isolates were *A. alternata* and nine were *A*. *arborescens* (Figure 2). It should also be noted that two ex-type species missing from the phylogenetic analysis, *A*. *doliconidium* and *A*. *italica*, are closely related to *A. alternata* (Li et al. 2022). Thus, more sequences from *A*. *doliconidium* and *A*. *italica* need to be obtained before conclusively identifying the species designations of this group of 15 *Alternaria* isolates. The remaining *Alternaria* isolates, except for *Alternaria* 7C, have good bootstrap evidence, 82, for being *A. ovoidea*. Additional sequences are needed for species identification of *Alternaria* 7C.

Li J, Phookamsak R, Jiang H, Bhat DJ, Camporesi E, Lumyong S, Kumla J, Hongsanan S, Mortimer PE, Xu J, Suwannarach N (2022) Additions to the inventory of the genus Alternaria section Alternaria (Pleosporaceae, Pleosporales) in Italy. J Fungi 24: 898

Figure 2. Neighbor joining tree of *Alternaria* species reconstructed from a multilocus gene alignment. The phylogenetic analysis utilized partial sequences of three genes (*Alt-a1, gapdh,* and *rpb2*). Type organisms in the phylogenetic tree were noted in the following manner: “A species name”. *Alternaria* organisms from this study were noted as numbers or a combination of numbers and letters. Bootstrap values were based on 3000 pseudoreplicates as indicated on the branch points. The scale bar corresponds to 0.01 nucleotide substitutions per site.


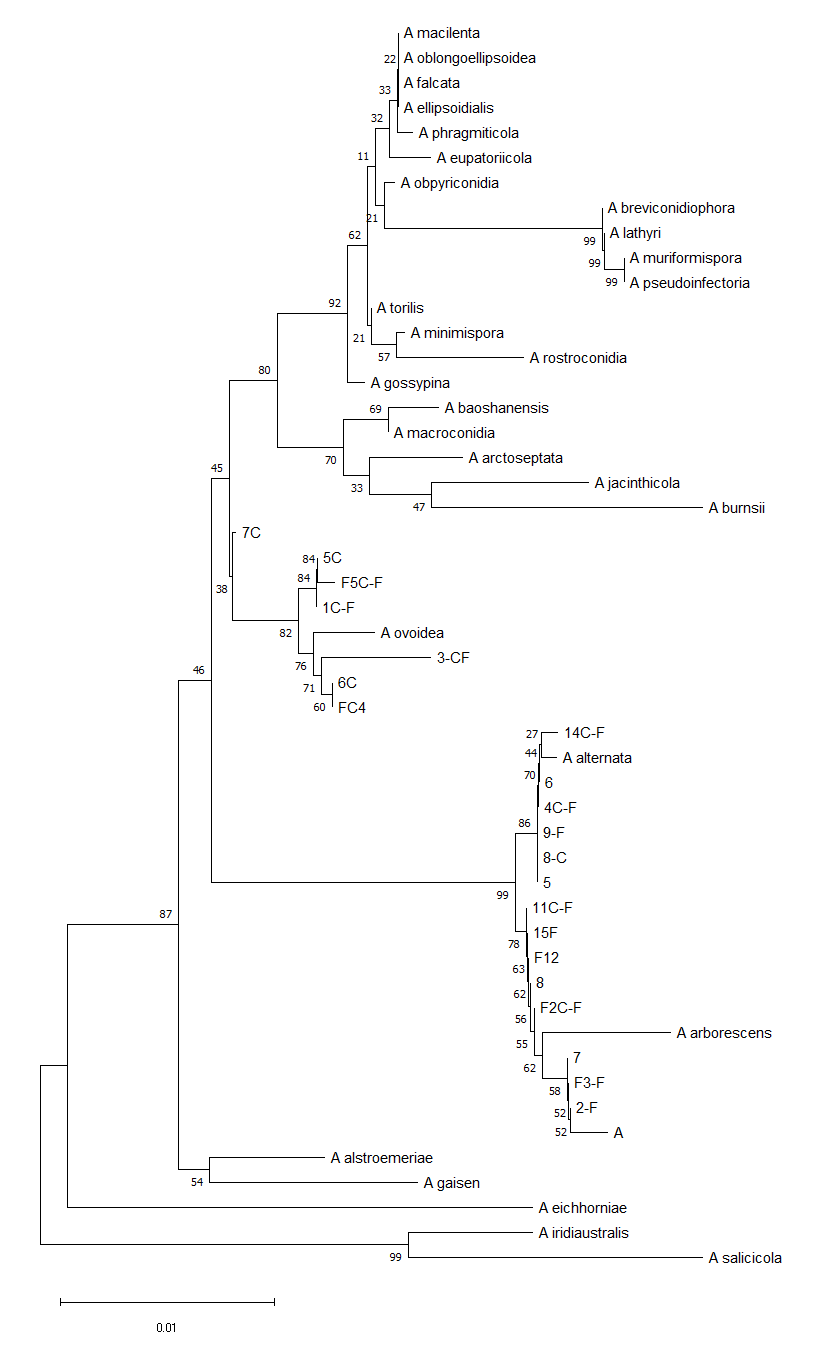

Supplement: Supplementary file 1 [file microorganisms-11-01550-s001.zip › Supplementary file S3.docx]
