# Supplementary material for: Potential Biocontrol Agents of Corn Tar Spot Disease Isolated from Overwintered Phyllachora maydis Stromata
Source: Microorganisms. 2023 Jun 10;11(6):1550. doi: 10.3390/microorganisms11061550 (PMC10303863; doi:10.3390/microorganisms11061550)
Supplement: Supplementary file 1 [file microorganisms-11-01550-s001.zip › Supplementary file S4.pptx]

## Slide 1
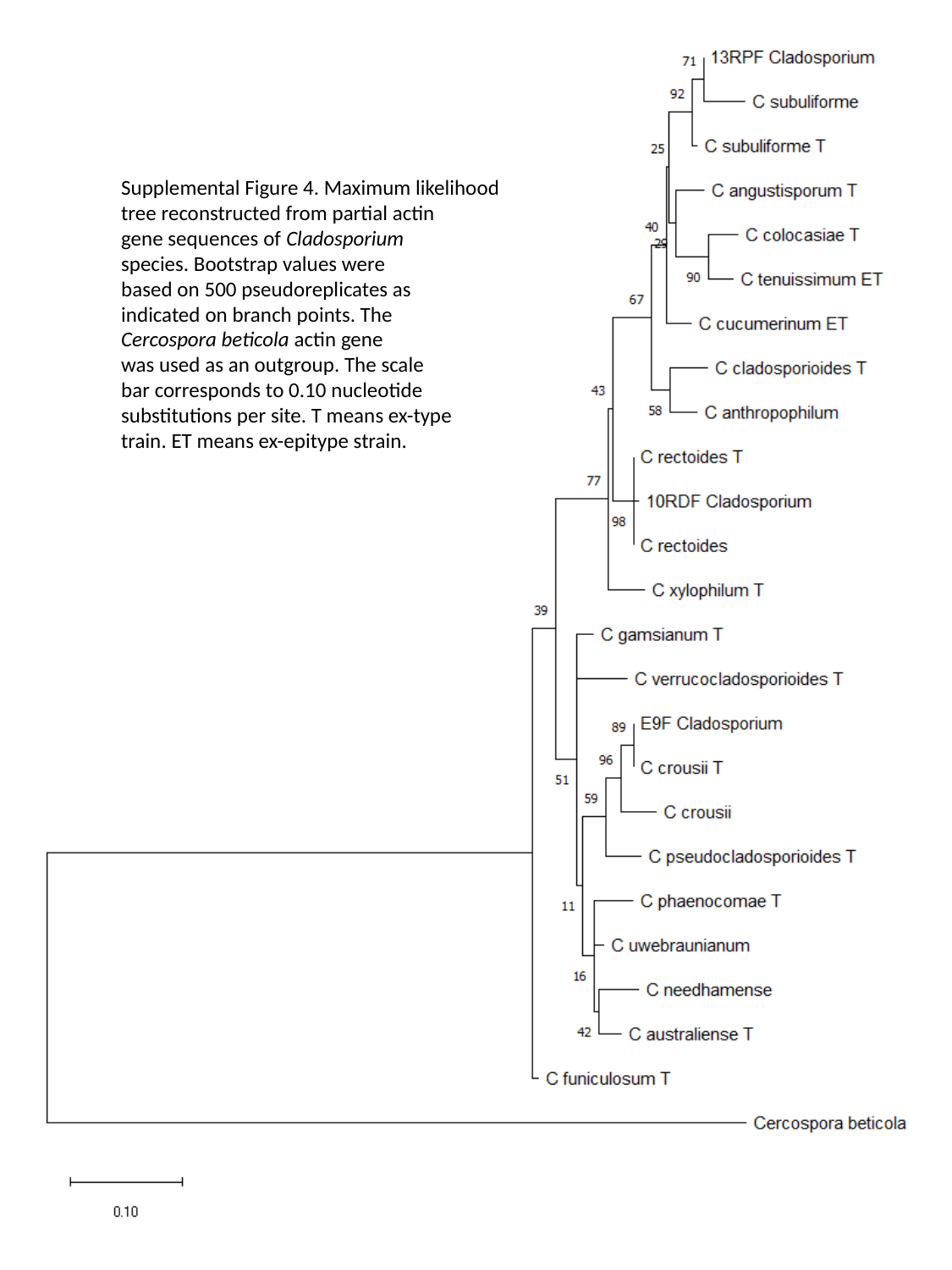

Supplemental Figure 4. Maximum likelihood
tree reconstructed from partial actin
gene sequences of Cladosporium
species. Bootstrap values were
based on 500 pseudoreplicates as
indicated on branch points. The
Cercospora beticola actin gene
was used as an outgroup. The scale
bar corresponds to 0.10 nucleotide
substitutions per site. T means ex-type
train. ET means ex-epitype strain.
